# Supplementary material for: Spatiotemporal atlas of pro-inflammatory NF-κB and anti-inflammatory STAT6 signaling using reporter mice during mRNA vaccination
Source: Mol Ther. 2026 Jun 2;34(8):4998–5014. doi: 10.1016/j.ymthe.2026.05.028 (PMC13464299; doi:10.1016/j.ymthe.2026.05.028)
Supplement: Supplementary file 1 [file mmc1.pdf]

YMTHE, Volume *34*

Supplemental Information

Spatiotemporal atlas of pro-inflammatory NF-κB and anti-inflammatory STAT6 signaling using reporter mice during mRNA vaccination

Electra Brunialti, Alessia Panzeri, Nicoletta Rizzi, Alessandro Villa, Clara Meda, Chiara Sfogliarini, Stefano Persano, Federico Arlati, Maria L. Guevara Lopez, Cristina Martelli, Gaetano Cannavale, Monica Rebecchi, Clara Di Vito, Letizia Venturini, Luisa Ottobrini, Domenico Mavilio, Susanna Fagerholm, Saverio Minucci, Elisabetta Vegeto, and Paolo Ciana

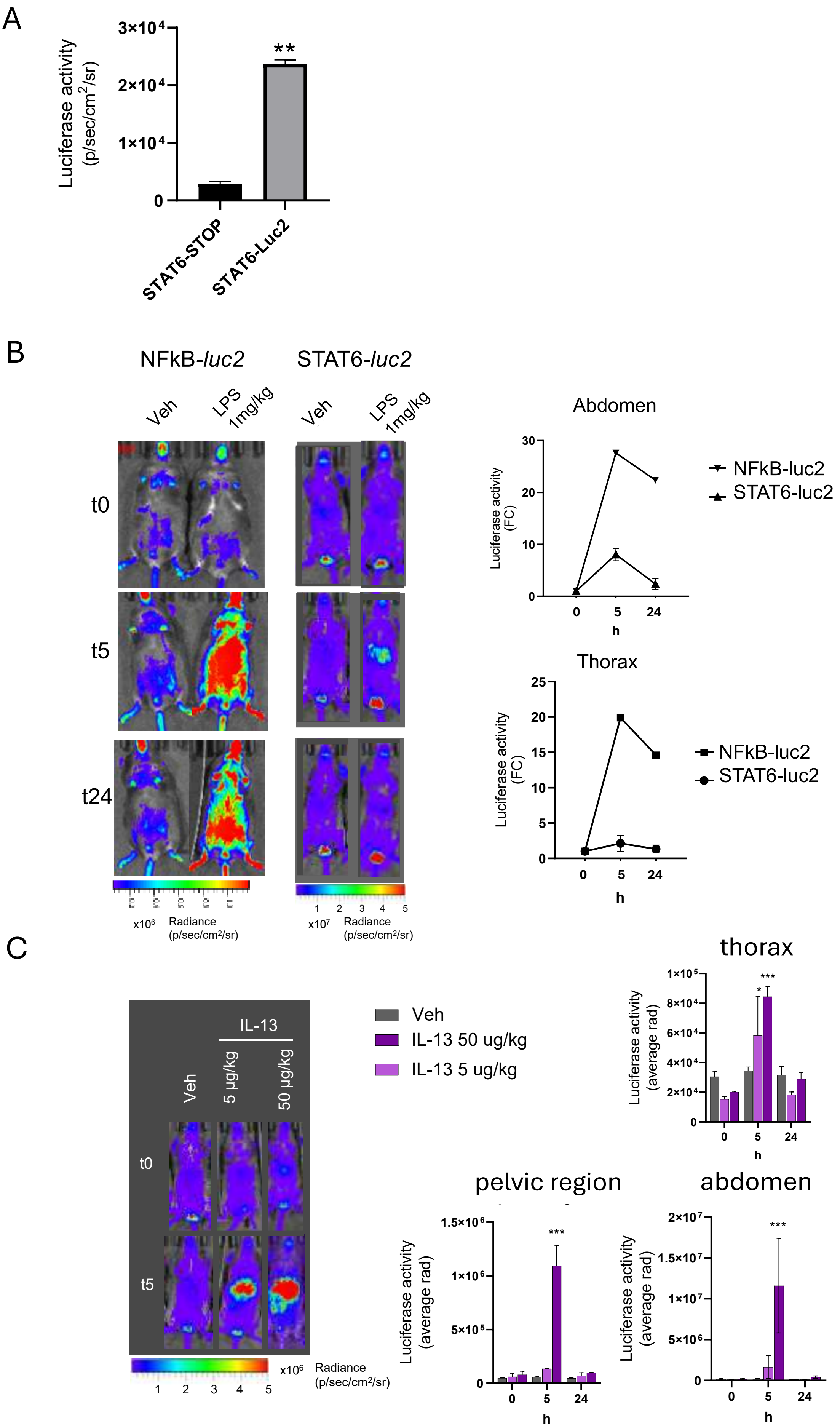
A
Luciferase activity (p/sec/cm2/sr)
3×10⁴
2×10⁴
1×10⁴
0
**
STAT6-STOP
STAT6-Luc2
B
NFkB-luc2
STAT6-luc2
Veh
LPS 1mg/kg
t0
t5
t24
x10⁶ Radiance (p/sec/cm²/sr)
1 2 3 4 5
x10⁷ Radiance (p/sec/cm²/sr)
Abdomen
Luciferase activity (FC)
30
20
10
0
0 5 24
h
NFkB-luc2
STAT6-luc2
Thorax
25
20
15
10
5
0
C
IL-13
Veh
5 µg/kg
50 µg/kg
x10⁶ Radiance (p/sec/cm²/sr)
Veh
IL-13 50 ug/kg
IL-13 5 ug/kg
thorax
Luciferase activity (average rad)
1×10⁵
8×10⁴
6×10⁴
4×10⁴
2×10⁴
*
***
pelvic region
1.5×10⁶
1×10⁶
5×10⁵
abdomen
2×10⁷
1.5×10⁷
1×10⁷
5×10⁶

Figure S1. STAT6 reporter mouse characterization. (A) Quantification of whole-body bioluminescence (BLI) in STAT6-STOP and STAT6-*luc2* mice. Data are presented as total luciferase activity (n = 2). Statistical significance was assessed using an unpaired *t*-test (**$p < 0.01$). **(B)** *in vivo* BLI of NFκB-*luc2 and* STAT6-*luc2* mice treated with LPS (1 mg/kg, intraperitoneally) at 0, 5, and 24 hours post-injection. Representative images (left) and corresponding quantification (right) are shown. Luciferase activity is expressed as fold change relative to baseline (t0). **(C)** In vivo bioluminescence imaging of STAT6-*luc2* mice following intraperitoneal administration of IL-13 (5 or 50 µg/kg). BLI was quantified in defined regions of interest (Figure 2) and is shown together with representative images. Statistical analysis was performed using two-way ANOVA followed by Dunnett's test versus vehicle (*$p < 0.05$, ***$p < 0.001$).

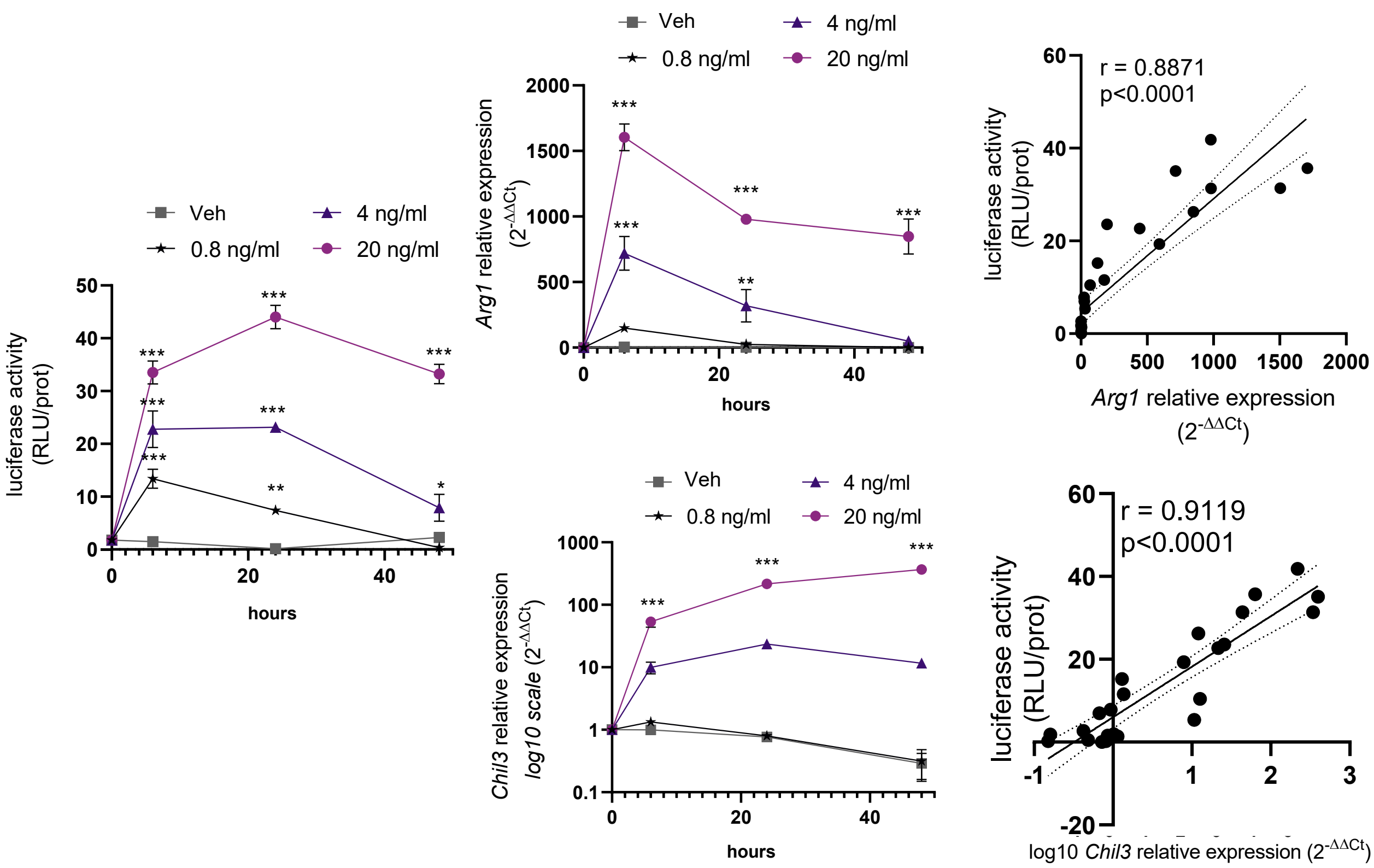

Figure S2. IL-4–induced responses in bone marrow–derived macrophages. Bone marrow–derived macrophages were treated with IL-4 at the indicated doses (0, 0.8, 4, 20 ng/mL) for 0, 6, 24, or 48 h. Luciferase activity in protein extracts is shown as RLU per µg protein (mean ± SEM). Relative expression of *Arg1* and *Ym1* (*Chil3*) is shown (*Arg1*, upper panels; *Ym1*, lower panels; mean ± SEM). Statistical analysis was performed using two-way ANOVA with Dunnett's test versus vehicle (n = 2). Correlation between luciferase activity and gene expression is shown (right); Pearson's r and p-values are reported.

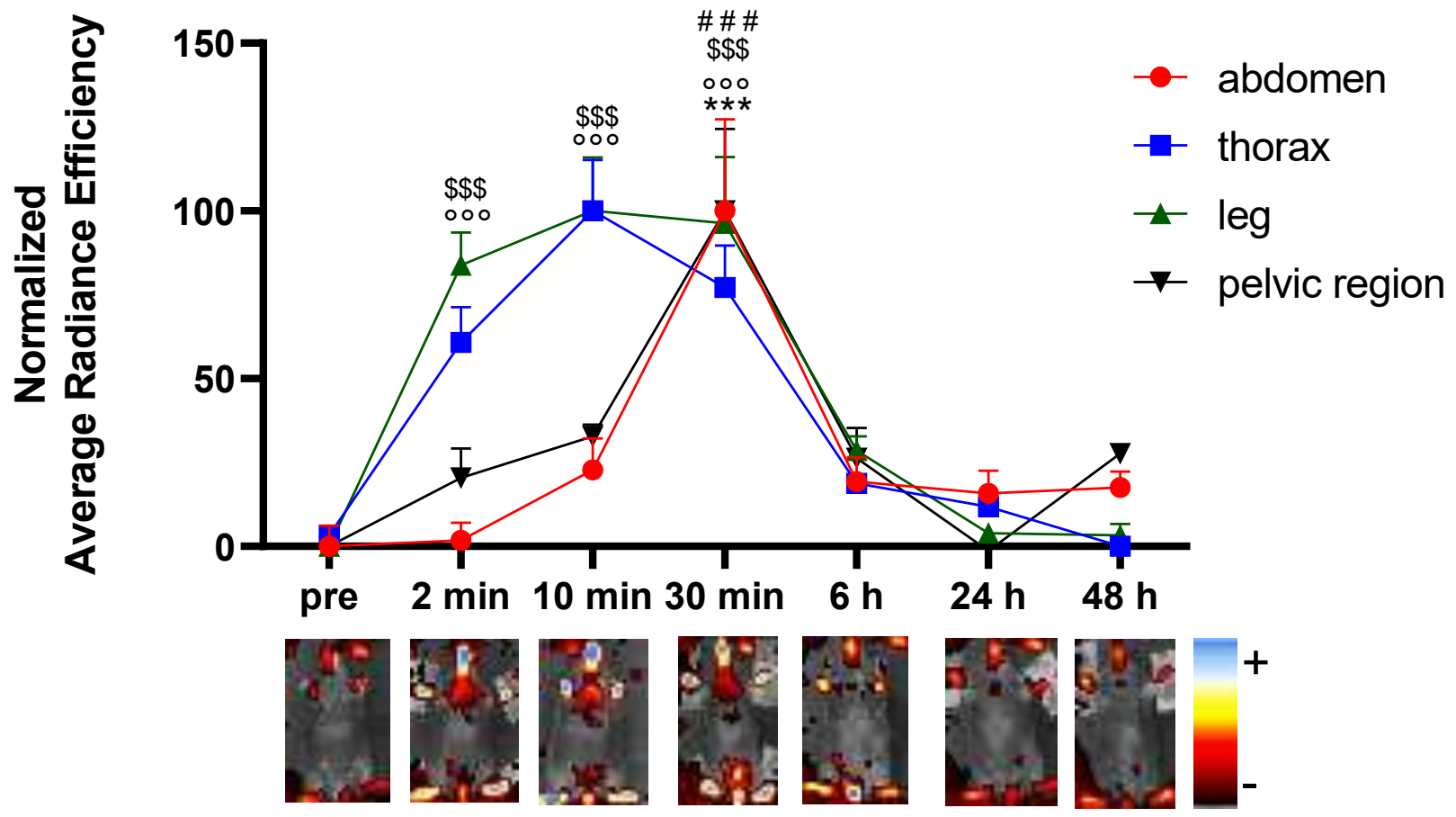

Figure S3. **Kinetic profile of fluorescently labelled mRNA-LNPs *in vivo* in different body districts**. C57BL/6 mice were intravenously injected with 10 µg of Cy5.5-labelled m¹Ψ-modified mRNA. Fluorescence was monitored at serial time points by *in vivo* imaging. Normalized fluorescence signals are presented as the mean ± SEM per mouse for each specified region of interest (ROI). For each body district, the highest detected signal has been placed at 100%. Representative *in vivo* images are shown. Statistical analysis was performed using two-way ANOVA followed by Dunnett's multiple comparisons test versus time zero (pre). $$$ $p < 0.001$ (leg); °°° $p < 0.001$ (thorax); *** $p < 0.001$ (abdomen); ### $p < 0.001$ (pelvic region).

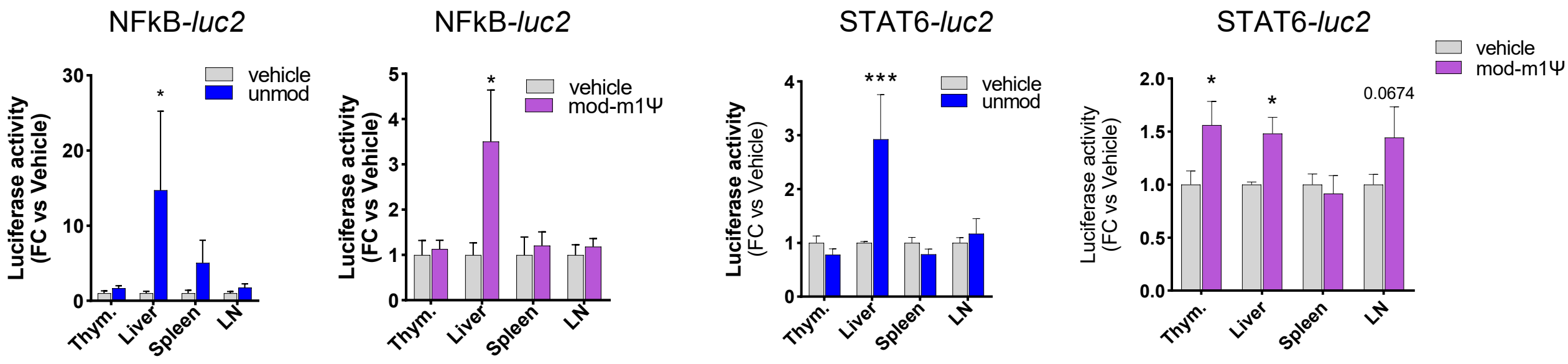

Figure S4. Bioluminescence in immune organs 5 hours after systemic vaccine administration. Bioluminescence emission from selected immune organs is shown 5 h after treatment. Quantification (derived from Figure 5A) is presented as mean photon emission (photons/s/cm²/sr) normalized to vehicle ± SEM. Statistical analysis was performed using two-way ANOVA with Dunnett's test versus vehicle (*p < 0.05, ***p < 0.001). Thym, thymus; LN, inguinal lymph nodes (right and left).

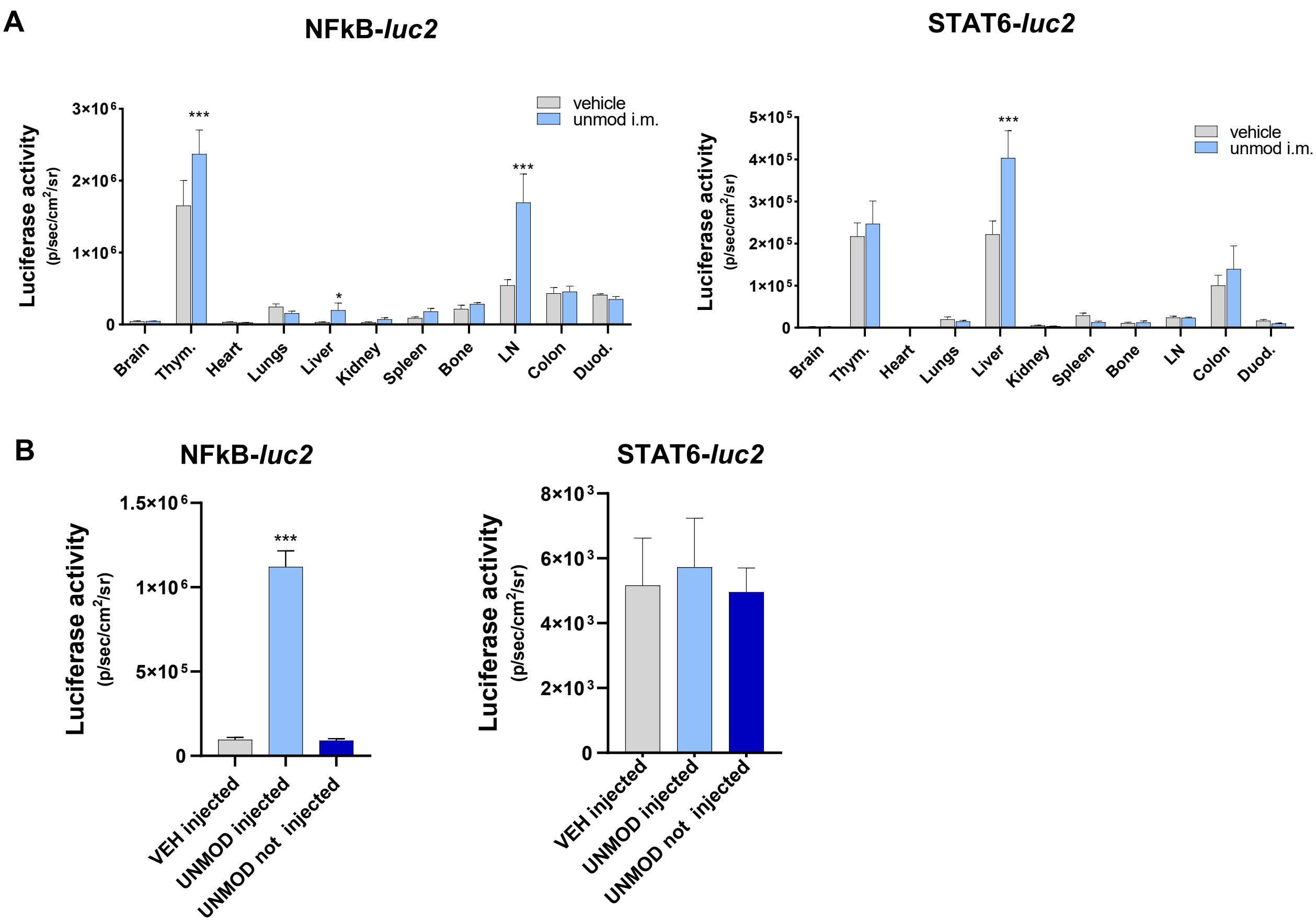

Figure S5. Ex vivo bioluminescence 5 hours after intramuscular administration of mRNA-LNP vaccine. Quantification of bioluminescence in explanted organs from NFκB-*luc2* and STAT6-*luc2* reporter mice 5 h after treatment with unmodified vaccine (10 µg) or vehicle (glucose solution). **(A)** Data are presented as mean photon emission (photons/s/cm²/sr) ± SEM (n = 6). Statistical analysis was performed using two-way ANOVA with Dunnett's test versus vehicle (*p < 0.05, **p < 0.01, ***p < 0.001). Thym, thymus; LN, inguinal lymph nodes (right and left); Duod., duodenum. **(B)** Bioluminescence signal in injected and contralateral (non-injected) muscle. Data are presented as mean photon emission (photons/s/cm²/sr) ± SEM (n = 3). Statistical analysis was performed using one-way ANOVA with Dunnett's test versus vehicle (***p < 0.001).

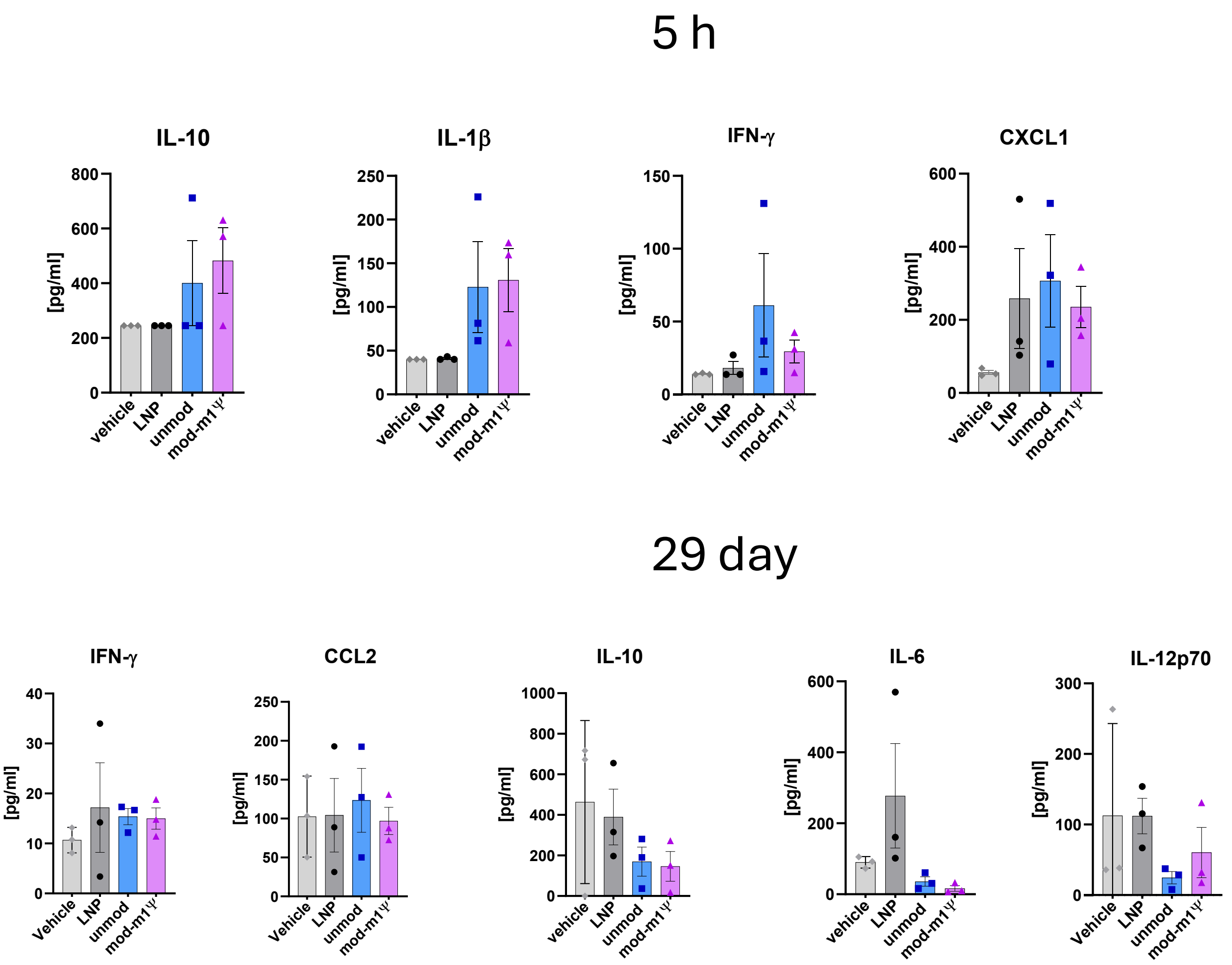

Figure S6. Plasma cytokine levels. Plasma cytokine levels at 5 hours and 29 days post-administration are shown. Data are presented as mean ± SEM (n = 3). Statistical analysis was performed using one-way ANOVA with Dunnett's test versus vehicle; no significant differences were detected. LNP: empty lipid nanoparticles; unmod: unmodified vaccine; mod-m1Ψ: N1-methylpseudouridine-modified vaccine.

A

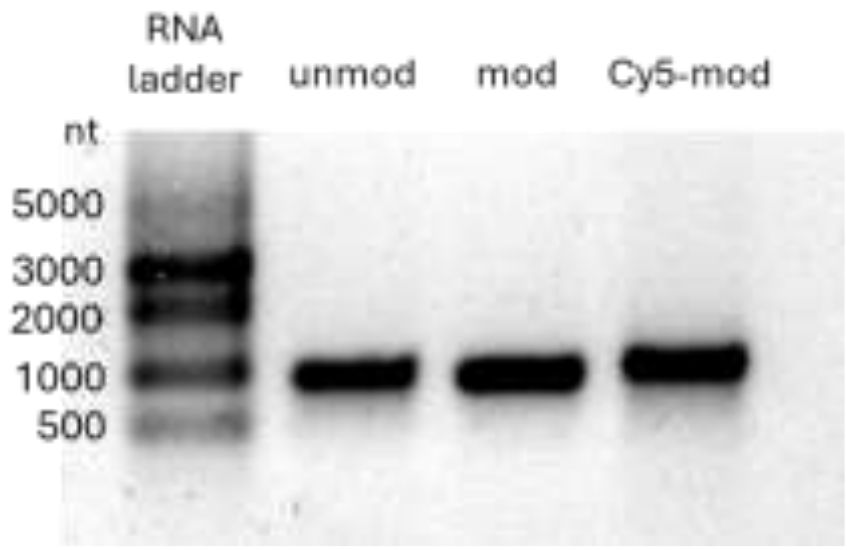

B

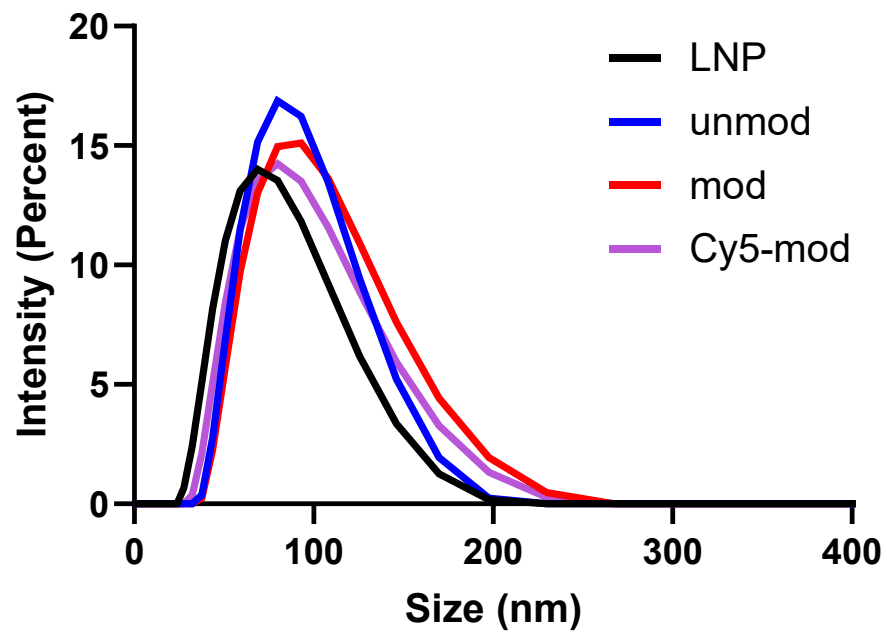

C

Formulation	Z-average (nm)	PDI	Zeta potential (mV)	EE(%)
**LNP**	70.22	0.2132	-2.525	N/A
**unmod**	82.96	0.1773	-6.255	93.2
**mod**	85.13	0.1111	-7.425	92.6
**Cy5-mod**	75.64	0.1459	-7.18	91.8

Figure S7. Physicochemical characterization of RNA-loaded lipid nanoparticles. (A) Agarose gel electrophoresis of synthesized mRNAs. An RNA ladder (nt) is shown alongside unmodified mRNA (unmod), N1-methylpseudouridine-modified mRNA (mod), and Cy5-labelled modified mRNA (Cy5-mod). **(B)** Size distribution profiles measured by dynamic light scattering (DLS) for LNPs encapsulating different RNA constructs. Curves correspond to empty LNPs (LNP, black), unmodified mRNA (unmod, blue), modified mRNA (mod, red), and Cy5-modified mRNA (Cy5-mod, purple). **(C)** Summary of physicochemical properties of LNPs formulations, including Z-average diameter, polydispersity index (PDI), zeta potential, and encapsulation efficiency (EE%).
