# Supplementary material for: Spatiotemporal atlas of pro-inflammatory NF-κB and anti-inflammatory STAT6 signaling using reporter mice during mRNA vaccination
Source: Mol Ther. 2026 Jun 2;34(8):4998–5014. doi: 10.1016/j.ymthe.2026.05.028 (PMC13464299; doi:10.1016/j.ymthe.2026.05.028)
Supplement: Supplementary file 2 [file mmc2.pdf]

Molecular Therapy
Original Article

Spatiotemporal atlas of pro-inflammatory NF-κB and anti-inflammatory STAT6 signaling using reporter mice during mRNA vaccination

Electra Brunialti,[1,12] Alessia Panzeri,[1,2,12] Nicoletta Rizzi,[3] Alessandro Villa,[1] Clara Meda,[1] Chiara Sfogliarini,[4] Stefano Persano,[5,6] Federico Arlati,[5,6] Maria L. Guevara Lopez,[5,6] Cristina Martelli,[7] Gaetano Cannavale,[7] Monica Rebecchi,[1] Clara Di Vito,[8,9] Letizia Venturini,[8] Luisa Ottobrini,[7,10] Domenico Mavilio,[8,9] Susanna Fagerholm,[11] Saverio Minucci,[5,6] Elisabetta Vegeto,[4] and Paolo Ciana[1]

[1]Department of Health Sciences, University of Milan, 20142 Milan, Italy; [2]Department of Pharmacological and Biomolecular Sciences Rodolfo Paoletti, University of Milan, 20133 Milan, Italy; [3]Animal Care Unit, University of Milan, 20133 Milan, Italy; [4]Department of Pharmaceutical Science, University of Milan, 20133 Milan, Italy; [5]Department of Oncology and Hematology-Oncology, University of Milan, 20122 Milan, Italy; [6]Department of Experimental Oncology, European Institute of Oncology IRCCS, 20141 Milan, Italy; [7]Department of Pathophysiology and Transplantation, University of Milan, 20122 Milan, Italy; [8]Department of Medical Biotechnologies and Translational Medicine (BioMeTra), University of Milan, 20090 Segrate, Italy; [9]IRCCS Humanitas Research Hospital, Rozzano, 20089 Milan, Italy; [10]National Research Council, Institute of Molecular Bioimaging and Physiology, 20054 Milan, Italy; [11]Faculty of Biological and Environmental Sciences, University of Helsinki, 00014 Helsinki, Finland

Immunization engages a highly coordinated series of innate and adaptive immune responses across multiple anatomical sites, a complexity that has contributed to the predominantly empirical nature of vaccine development. To address this limitation, we developed and validated a STAT6 reporter mouse that enables dynamic whole-body imaging and *ex vivo* characterization of STAT6-mediated anti-inflammatory signaling. We then integrated this model with an established NF-κB reporter system to dissect the immune activation triggered by two lipid nanoparticle (LNP)-formulated mRNA vaccines differing in RNA chemistry (unmodified versus N^1-methylpseudouridine-modified). The dual-reporter approach enabled the construction of a spatiotemporal atlas of vaccine-induced signaling, revealing distinct immune dynamics driven by RNA chemistry and identifying the liver as an early hub for both NF-κB and STAT6 activity following systemic administration. Correlation with serological data showed that early STAT6 activation followed by rapid signal resolution was associated with favorable humoral responses. These findings establish NF-κB and STAT6 reporter mice as rapid *in vivo* screening tools for early evaluation of vaccine immune potential and highlight their ability to inform more mechanistic and rational design of mRNA vaccine platforms, while emphasizing the role of the liver both as a primary LNP target and as an immunologically relevant organ.

INTRODUCTION

The immunization process unfolds through a highly coordinated sequence of molecular and cellular events that progressively recruit innate and adaptive immune components across distinct anatomical niches.[1] Immune activation against antigens begins with rapid innate sensing followed by the staged engagement of adaptive mechanisms. Within minutes, antigens are detected by pattern-recognition receptors (PRRs) expressed on innate immune sentinels. This immediate detection triggers inflammatory signaling cascades, cytokine release, and recruitment of additional effector cells, creating the microenvironment required for subsequent adaptive priming. Over the next 6–24 h, antigen-presenting dendritic cells (DCs) undergo maturation and migrate to draining lymph nodes, where they present peptide-MHC complexes to naive $CD4^+$ and $CD8^+$ T cells while delivering the co-stimulatory signals required for activation. Within 1–3 days, T cells bearing cognate T cell receptors undergo metabolic and transcriptional reprogramming that initiates rapid clonal expansion.[2–4] Activated $CD4^+$ T cells provide cytokines such as interleukin (IL)-2 that amplify $CD8^+$ T cell expansion, cytotoxic differentiation, and trafficking toward sites of antigen presence. In parallel, activated B cells interact with $CD4^+$ T cells, receiving instructive signals mediated by CD40L, IL-4, and IL-21, which drive class switching and commitment either to early plasmablast differentiation or entry into germinal centers.[2–4] The primary antibody response becomes detectable within 4–7 days, initially dominated by IgM production, followed by class-switch recombination and affinity maturation. Within germinal centers, B cell clones undergo affinity-based selection, ultimately giving rise to long-lived plasma cells and memory B cells that sustain durable, high-affinity antibody production. After the primary response contracts over 2–4 weeks, antigen-specific

Received 4 February 2026; accepted 26 May 2026;
https://doi.org/10.1016/j.ymthe.2026.05.028.

[12]These authors contributed equally

Correspondence: Paolo Ciana, University of Milan, Via Antonio di Rudinì 8, 20142 Milan, Italy.
E-mail: paolo.ciana@unimi.it

 © 2026 The Author(s).
Published by Elsevier Inc. on behalf of The American Society of Gene and Cell Therapy.
This is an open access article under the CC BY license (http://creativecommons.org/licenses/by/4.0/).

memory T and B cells persist, providing a rapid and long-lasting response upon re-exposure.[5,6]

Accordingly, successful immunization depends on the coordinated and temporally ordered engagement of molecular and cellular programs, governed by transcriptional regulators and finely tuned intercellular signaling networks involving cytokines, chemokines, and co-stimulatory molecules. The spatial and temporal control of these pathways across distinct anatomical compartments is essential for generating robust and long-lasting protective immunity while limiting pathological inflammation and preserving immune homeostasis.[4] Within this regulatory landscape, NF-κB and STAT (Signal Transducer and Activator of Transcription) signaling pathways function as key hubs integrating inflammatory cues with lymphocyte activation, proliferation, and differentiation.[7,8]

NF-κB, a key mediator of pro-inflammatory responses, acts as an immediate-early transcription factor. It is activated within minutes of antigen sensing in peripheral tissues via PRR-dependent IκB degradation and nuclear translocation.[7] This early activity drives the production of inflammatory cytokines, chemokines, and co-stimulatory molecules that coordinate DC maturation.[9,10] In draining lymph nodes, sustained yet tightly regulated NF-κB activity over 6–24 h further fine-tunes T cell activation, thereby ensuring an effective adaptive immune response.[11,12]

In parallel, STAT proteins direct the polarization of distinct T helper (Th) cell subsets. Among them, STAT6 mediates over 80% of the genes regulated by the key anti-inflammatory cytokine IL-4, and it acts as a major driver of the transcriptional program defining Th2 differentiation.[13] STAT6 activation marks a later, instructive phase of adaptive immunity, driving IgE class switching and broader Th2-specific transcriptional responses,[14] while STAT6 signaling within B cells is essential for proper germinal center formation.[8] Importantly, dysregulation of NF-κB or STAT6 compromises both the magnitude and quality of immune responses, leading to insufficient T and B cell activation, impaired antibody production, and altered Th2 polarization, ultimately reducing vaccine efficacy or increasing the risk of excessive inflammation.[9,15,16]

Despite extensive knowledge of immunological mechanisms, most vaccines have been developed empirically,[4] and the spatiotemporal dynamics of NF-κB and STAT6 *in vivo* remain poorly defined. Clarifying when and where these pathways are engaged could guide the rational design of vaccines and adjuvants to optimize immunity while limiting harmful inflammation.

In this context, the use of *in vivo* murine reporter systems represents a powerful experimental approach, as it allows for the dynamic and non-invasive analysis of transcription factor activation in living organisms.[17] Leveraging this strategy, our lab has investigated the temporal and organ-specific functions of various transcription factors in the context of drug discovery and in the study of physiological and pathological conditions,[18–20] including studies on pro-inflammatory responses using the NF-κB reporter mouse.[21] However, the development of reporter systems capable of tracking complementary anti-inflammatory pathways would be essential to obtain a comprehensive view of immune regulatory processes.

To address these gaps, we first generated a reporter mouse model to investigate anti-inflammatory signaling mediated by STAT6 by optical *in vivo* imaging. We then developed *in vivo* imaging strategies that enable real-time, longitudinal monitoring of both NF-κB and STAT6 activation following immunization: we compared two mRNA vaccine constructs encoding the same antigen but differing in their chemical composition: an unmodified mRNA expected to strongly engage innate RNA sensors, and an N^1-methylpseudouridine ($m^1\Psi$)-modified mRNA designed to attenuate innate immune recognition while enhancing mRNA stability and translational efficiency.[22–24] By administering the vaccines intravenously to achieve systemic biodistribution, we generated a spatially and temporally resolved atlas of NF-κB and STAT6 activation across the body, providing a comprehensive framework to elucidate how vaccine chemistry influences immune signaling dynamics *in vivo*.

RESULTS

Generation of STAT6 reporter mouse lines

To enable real-time monitoring of the anti-inflammatory pathway mediated by STAT6, we developed a dual-reporter system designed to track STAT6 transcriptional activity both *in vivo* and *ex vivo*. The reporter construct was based on a bicistronic design comprising a firefly luciferase gene (*luc2*) for *in vivo* bioluminescence imaging and a green fluorescent protein (GFP) gene for single-cell analysis, separated by an internal ribosome entry site (IRES).[21]

Upstream of the bicistronic cassette, we cloned a synthetic promoter containing two tandem copies of the validated STAT6 response element from the *p2xSTAT6-luc2P* plasmid, generating the *p2xSTAT6-GFP-luc2* construct. The ability of this promoter configuration to report STAT6 activation was tested by transient transfection in RAW 264.7 macrophages. Cells were stimulated with cytokines known to activate STAT6-dependent transcription (IL-4 or IL-13, 20 ng/mL),[25] the anti-inflammatory cytokine IL-10 (20 ng/mL, acting through STAT3),[26] or the pro-inflammatory stimulus lipopolysaccharide (LPS, 2 μg/mL) (Figure 1A). Bioluminescence assays performed 24 h after cytokine exposure, or 6 h after LPS treatment, revealed a robust induction of luciferase activity following IL-4 and IL-13 stimulation, whereas IL-10 and LPS failed to elicit reporter activation. These results confirmed the specificity of the construct for STAT6-mediated signaling. The functionality of the GFP reporter was evaluated in HT-29 cells transiently transfected with the STAT6 construct together with a plasmid constitutively expressing tdTomato, used as a positive control for transfection efficiency. A clear GFP fluorescence was detected in tdTomato-positive cells following 24-h treatment with 20 ng/mL IL-4, whereas no significant GFP signal was observed in any cells treated with 100 ng/mL LPS or vehicle (water) (Figure 1B). These results demonstrate the proper functionality of both reporters. Encouraged by these results, we

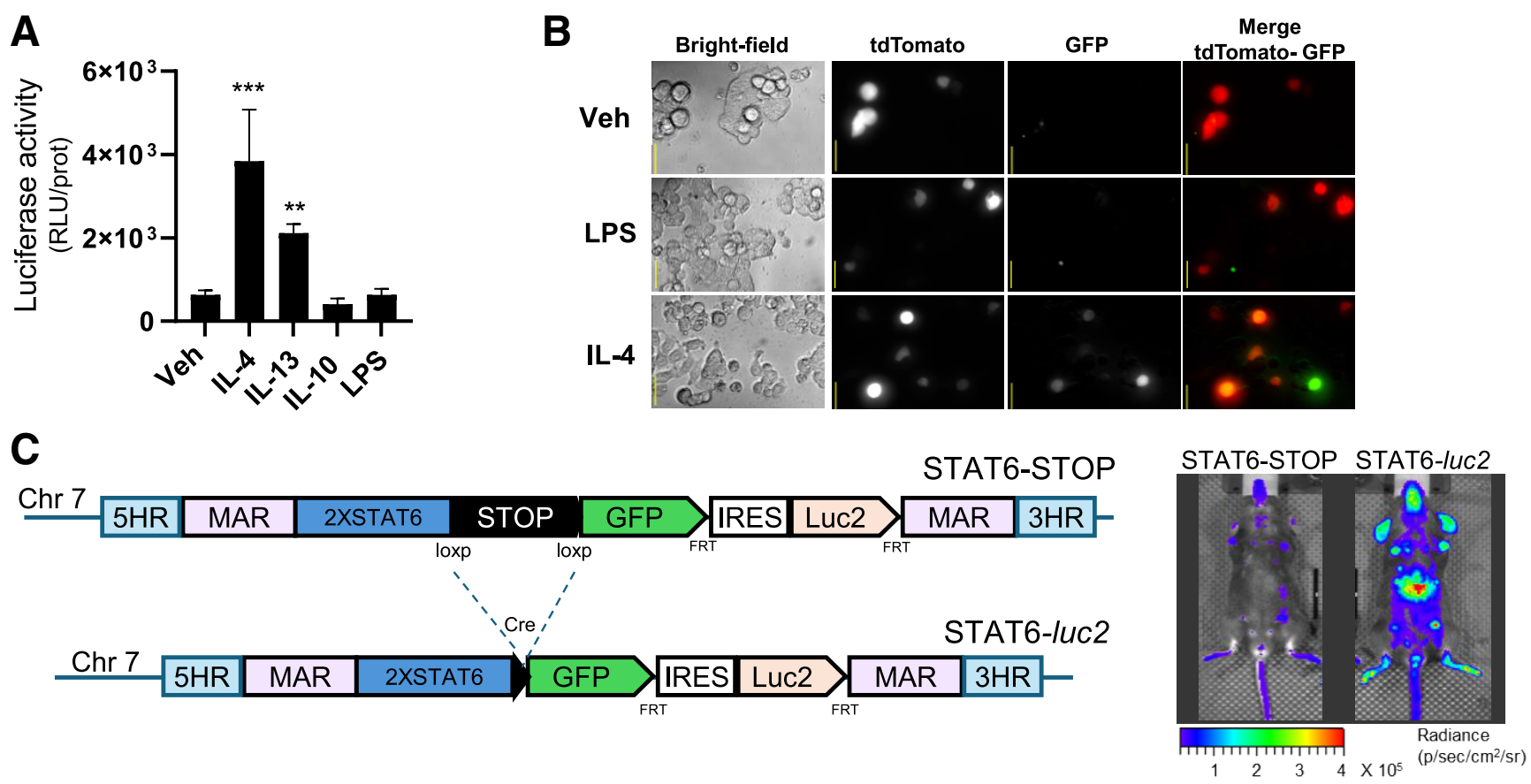

Figure 1. Design and functional analysis of STAT6 reporter systems

(A) Luciferase activity in RAW 264.7 cells transiently transfected with the *p2xSTAT6*-GFP-*luc2* construct and treated with 20 ng/mL cytokines for 24 h, 2 μg/mL LPS for 6 h, or vehicle (water). Data are mean ± SD (n = 4) of luciferase activity (RLU) normalized to total protein content. ***p < 0.001, **p < 0.01 versus vehicle (one-way ANOVA, Dunnett's test). (B) Representative fluorescence microscopy images of HT-29 cells co-transfected with the p2xSTAT6-GFP-luc2 construct and a plasmid constitutively expressing tdTomato and treated with 20 ng/mL IL-4, 100 ng/mL LPS, or vehicle (water) for 24 h. Scale bar: 50 μm. (C) Schematic representation of the STAT6-STOP and STAT6-*luc2* reporter mouse transgenes before and after Cre-mediated excision of the STOP cassette. Abbreviations: Cre, Cre recombinase*;* Chr7, chromosome 7; 5HR, 3HR, homologous regions for chromosomal integration; MAR, matrix attachment regions; 2XSTAT6, STAT6 promoter; STOP, POLR2 (RNA polymerase II) termination signal; *loxP*, locus of X-over P1; GFP, green fluorescent protein; *luc2*, optimized firefly luciferase 2; IRES, internal ribosome entry site; FRT, Flp recombination target sites. On the right, representative pseudocolor images of ventral luciferase emission from STAT6-STOP and STAT6-*luc2* female mice are shown according to the indicated scale bar.

next generated the STAT6 reporter mice to investigate the modulation of anti-inflammatory signaling *in vivo*. Following a previously validated knock-in strategy routinely used in our laboratory, the reporter cassette was inserted into a defined transcriptionally active locus on mouse chromosome 7, which we have shown to support stable, position-independent expression.[21] To insulate the transgene from chromatin position effects, the construct was flanked by matrix attachment region (MAR) sequences derived from the chicken lysozyme gene (Figure 1C).[27] In addition, the reporter cassette was preceded by a floxed STOP sequence (RNA polymerase II termination signal), allowing tissue-specific activation upon Cre-mediated recombination. Embryonic stem cells harboring the correctly integrated construct were injected into blastocysts to generate chimeric mice carrying the floxed transgene, hereafter referred to as STAT6-STOP (Figure 1C). As expected, STAT6-STOP mice exhibited very low basal luciferase activity during *in vivo* imaging, confirming effective transcriptional blockade by the STOP cassette (Figures 1C and S1A). To obtain a constitutively active reporter line, STAT6-STOP mice were crossed with *B6.C-Tg(CMV-cre)1Cgn/J* mice, in which Cre recombinase is expressed in germ cells.[28] Germline excision of the floxed STOP sequence resulted in permanent activation of the reporter allele, which was transmitted to the progeny independently of Cre expression. Subsequent breeding with wild-type C57BL/6 mice eliminated the Cre transgene, yielding the STAT6-*luc2* line (Figure 1C). Consistent with the physiological activity of the transcription factor,[13,29] STAT6-*luc2* mice displayed robust and widespread bioluminescent signals throughout the body (Figures 1C and S1A), confirming that germline removal of the STOP cassette effectively restored reporter expression across all tissues.

Validation of the STAT6-*luc2* reporter mouse

To validate the functionality of the *STAT6-luc2* reporter line, we assessed whether luciferase expression could be induced following activation of STAT6-dependent anti-inflammatory signaling. Mice were intraperitoneally injected with either IL-4 (5 or 50 μg/kg), LPS (50 μg/kg or 1 mg/kg), or vehicle (PBS). Whole-body bioluminescence imaging was performed at 0, 5, and 24 h post-injection to monitor the kinetics of pathway activation (Figure 2A). Bioluminescent imaging revealed a clear, dose-dependent increase in photon emission in the abdominal and pelvic regions 5 h after IL-4 administration, with a moderate signal also detected in the thoracic area (Figure 2B). In contrast, LPS induced only a weak and non-dose-dependent increase in luminescence, confined mainly to the abdominal region and not reaching statistical significance. At 24 h post-administration, luciferase activity returned to baseline levels across all treatment groups, consistent with the transient kinetics typically associated with cytokine-driven STAT6 activation.[13,30] Comparable responses were observed upon intraperitoneal administration of IL-13, another canonical activator of STAT6 (Figure S1). Notably, under identical experimental conditions, LPS administration (1 mg/kg) elicited a robust inflammatory response in NFκB-*luc2* reporter mice, a reporter mouse model previously generated and validated by our lab,[21] whereas STAT6-*luc2* mice displayed markedly lower signal intensity (Figures 2A and 2B; S1). These findings confirm the pathway specificity of the STAT6 reporter and its selective responsiveness to anti-inflammatory-mediated signaling.

To further characterize tissue-specific reporter activation, *ex vivo* bioluminescence imaging was performed 5 h after intraperitoneal IL-4 injection (50 μg/kg). A widespread increase in bioluminescence was observed across multiple organs, with the most pronounced activation detected in the liver, stomach, duodenum, colon, and lung (Figures 2C and 2D) when compared with vehicle-treated mice. Consistent with these results, fluorescent optical imaging of liver tissue revealed detectable GFP signal in IL-4-treated animals, confirming GFP reporter expression (Figures 2E and 2F).

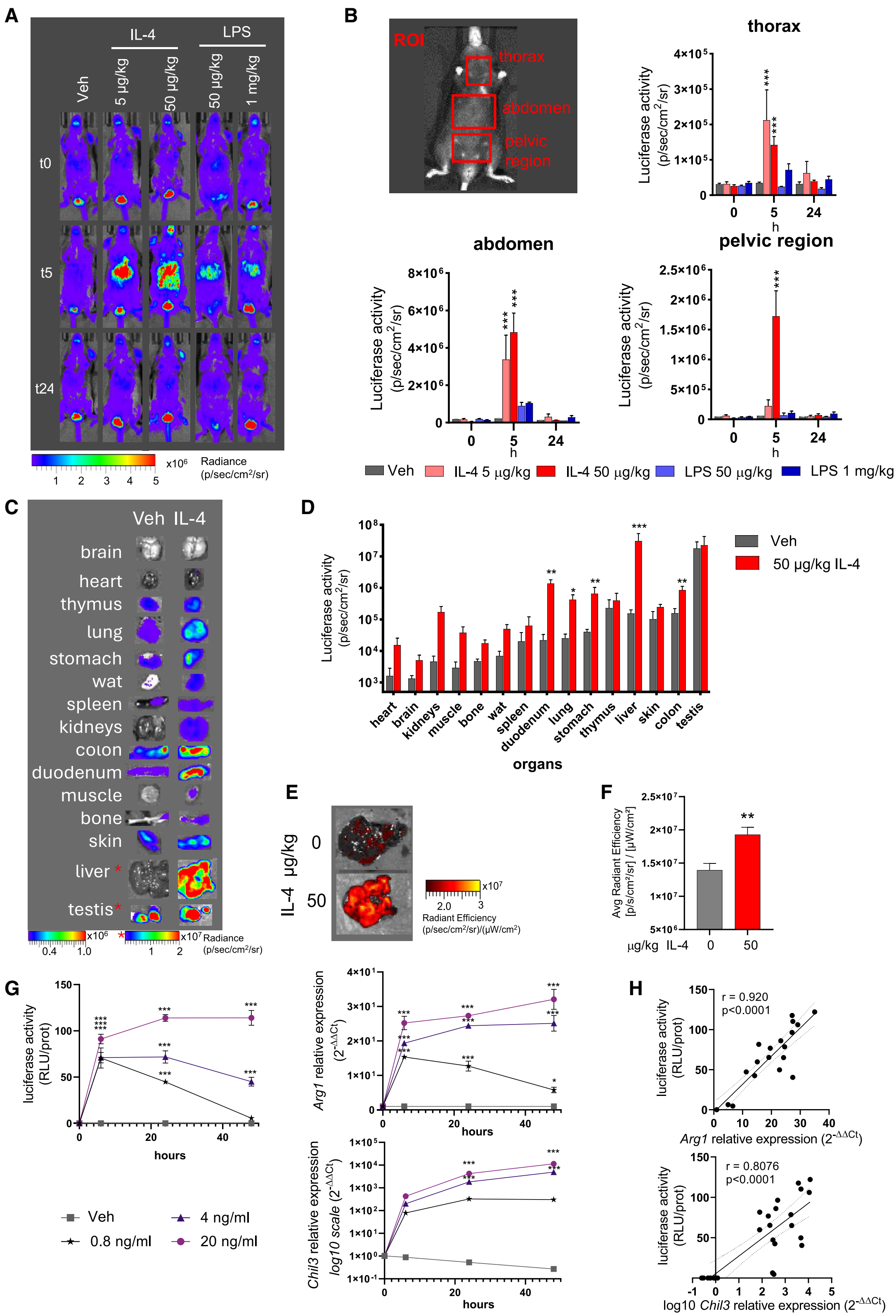

(legend on next page)

Finally, we sought to determine whether STAT6-*luc2* mice could also serve as a source of primary cells capable of reporting STAT6 activation: peritoneal macrophages were isolated from STAT6-*luc2* reporter mice and stimulated *in vitro* with increasing concentrations of IL-4 (0, 0.8, 4, and 20 ng/mL). Luciferase activity was measured at 0, 6, 24, and 48 h post-stimulation and compared with the expression levels of canonical STAT6 target genes *Arg1* and *Chil3* (also known as *Ym1*).[31] Luciferase activity closely paralleled *Arg1* expression, displaying a strong positive correlation between reporter induction and *Arg1* mRNA levels (Figures 2G and 2H). A similar trend was observed for *Chil3*, although the correlation followed a logarithmic rather than linear relationship. Comparable results were obtained using bone-marrow-derived macrophages (Figure S2).

Collectively, these data demonstrate that the STAT6-*luc2* reporter mouse is a reliable reporter of anti-inflammatory responses and can be used to monitor bioluminescence dynamics *in vivo*, at the tissue level *ex vivo*, and in primary cell cultures.

Reporter mouse models reveal distinct spatiotemporal modulation of pro- and anti-inflammatory pathways following mRNA vaccination

To elucidate how mRNA-based vaccination modulates pro- and anti-inflammatory responses over time and across distinct anatomical sites, we used two mRNA vaccine formulations encoding the same non-viral antigen derived from an orthologous gene. One vaccine contained an unmodified mRNA sequence (unmod), while the other incorporated N^1-methylpseudouridine (mod-m1Ψ) as a chemical modification aimed at reducing innate immune recognition. Both formulations were encapsulated in lipid nanoparticles (LNPs) with identical lipid composition. To assess their immunostimulatory potential, both mRNA formulations were tested *in vitro* using bone-marrow-derived dendritic cells (BM-DCs) from wild-type C57BL/6 mice. Cells were treated with 2.5 μg of mod-m1Ψ or unmod mRNA vaccines, their respective empty LNPs controls (LNP), or vehicle (water), for 24 and 48 h. Dendritic cell maturation was assessed by flow cytometric analysis of CD80, CD86, CD40, CCR7, and MHC-II expression, and IL-12 secretion was quantified by ELISA (Figure 3). After 24 h, only the unmodified mRNA molecule induced a pronounced upregulation of all surface maturation markers. At 48 h, both mRNA formulations promoted the upregulation of CD80, CD86, CD40, and CCR7; however, MHC-II expression and IL-12 secretion remained significantly higher in DCs treated with the unmodified vaccine. Neither the empty LNPs nor the vehicle controls induced detectable activation. These data indicate that both mRNA vaccines can promote DC maturation; however, the unmodified sequence elicits a more rapid response and exhibits greater immunostimulatory activity than its modified counterpart.

We next moved to an *in vivo* study, first assessing the kinetic profile of labeled mRNA–LNPs following tail intravenous administration. C57BL/6 mice received 10 μg of Cy5.5-labeled, $m^1\Psi$-modified mRNA, based on the assumption that biodistribution is primarily dictated by the LNP carrier rather than RNA chemistry.[32] The formulation exhibited rapid systemic distribution, with marked accumulation in the abdominal/pelvic region within 30 min, followed by fast clearance (Figure S3), consistent with the expected biodistribution of intravenously administered LNP-based formulations.[33]

Subsequently, to investigate the spatial and temporal modulation of inflammatory pathways, NFκB-*luc2* and STAT6-*luc2* reporter mice were injected intravenously (10 μg, tail vein) on day 0 with either mod-m1Ψ or unmod mRNA vaccines, or with the corresponding empty LNPs (LNP) as controls, followed by a booster dose on day 14. Bioluminescence imaging of reporter mice was performed at 0 h, 5 h, 1 day, and 6 days after each injection, and again on day 29 (14 days after the booster) to monitor reporter activation across different anatomical regions (Figure 4).

In NFκB-*luc2* mice (Figure 4A), the unmod vaccine induced a robust and rapid bioluminescent signal in the abdominal region as early as 5 h after the first and booster administrations, followed by prominent activation in the pelvic region at 1 day after both administrations. A mild but sustained increase in signal in the thoracic region was observed in mice receiving the unmodified vaccine from 5 h post-administration (day 0.2). In contrast, the mod-m1Ψ vaccine elicited a modest increase in the pelvic signal on day 1 and again from days 13 to 29, although these changes did not reach statistical significance. In STAT6-*luc2* mice (Figure 4B), both mRNA vaccines induced, in the abdominal region, a detectable activation of the anti-inflammatory pathway 5 h after the booster injection, and the unmod mRNA also elicited activation 5 h after the primary dose; the signal was consistently stronger in mice receiving the unmod vaccine.

Figure 2. Validation of STAT6-*luc2* reporter mice

(A) Representative *in vivo* bioluminescence imaging of STAT6-*luc2* reporter mice following intraperitoneal injection of IL-4 (5 or 50 μg/kg), LPS (0.05 or 1 mg/kg), or vehicle (veh, PBS). Images were acquired at 0, 5, and 24 h post-treatment. Pseudocolor scale indicates bioluminescence intensity. (B) Quantification of *in vivo* bioluminescent signal in selected regions of interest (ROIs, red squares). Bars represent mean photon flux (photons/s/cm^2/sr) ± SD ($n = 3$). ***$p < 0.001$ versus vehicle (two-way ANOVA, Dunnett's test). (C) Representative pseudocolor images of bioluminescence emitted from dissected organs collected 5 h after IL-4 (50 μg/kg) or vehicle (veh, PBS) administration and (D) quantification of *ex vivo* organ bioluminescence. Bars represent mean photon emission (photons/s/cm^2/sr) ± SD ($n = 4$). *$p < 0.05$, **$p < 0.01$, ***$p < 0.001$ versus vehicle (two-way ANOVA, Sidak's test). wat, white adipose tissue. (E) Representative pseudocolor images of fluorescence emitted from the liver 5 h after IL-4 (50 μg/kg) or vehicle treatment, and (F) quantification of liver fluorescence intensity. Bars represent average radiant efficiency [(p/s/cm^2/sr)/(μW/cm^2)] ± SD ($n = 3$). **$p < 0.01$ versus vehicle, determined by unpaired *t* test. (G) Peritoneal macrophages derived from STAT6-*luc2* mice were treated with IL-4 at different doses (0, 0.8, 4, 20 ng/mL) for 0, 6, 24, or 48 h. Luciferase expression in protein extracts is expressed as relative light unit (RLU) per μg of protein ±SEM. At the same time points, relative quantification of target gene transcripts (*Arg1* and *Chil3*) was performed using the $2^{-\Delta\Delta Ct}$ method versus vehicle ±SEM. ***$p < 0.001$ versus vehicle (two-way ANOVA, Dunnett's test). (H) Correlation between luciferase activity and gene relative expression reported in (G); Pearson r and *p* values for each interpolated curve are reported.

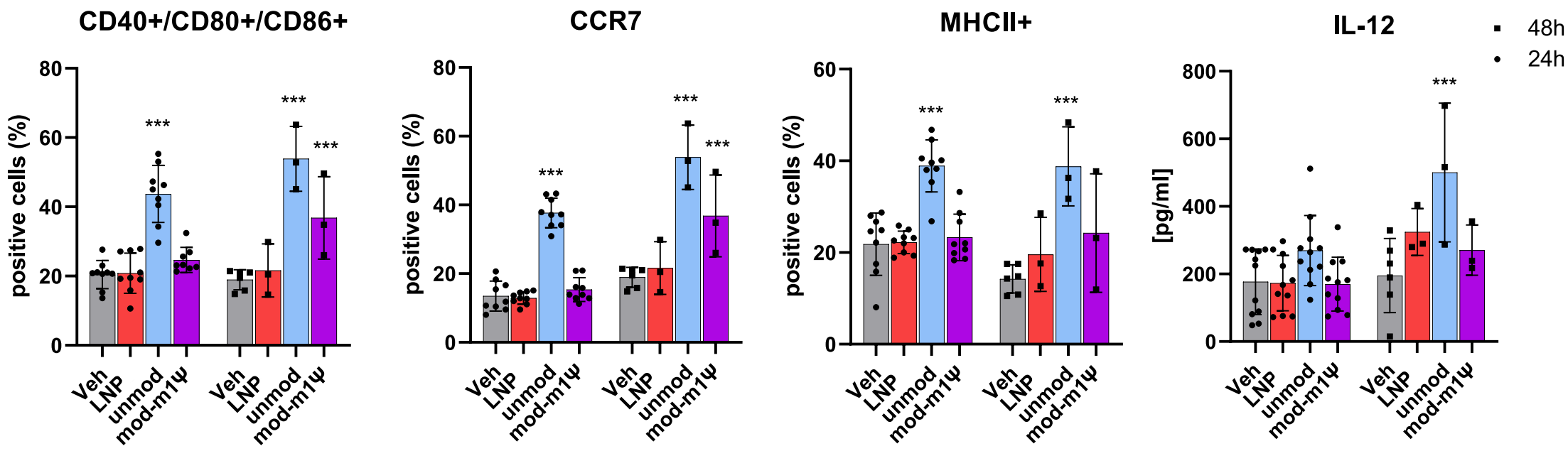

Figure 3. Maturation of bone-marrow-derived dendritic cells in response to mRNA-LNP treatment
Bone-marrow-derived dendritic cells were treated with 2.5 μg of modified (mod-m1Ψ) or unmodified (unmod) mRNA encapsulated in LNPs, with an equivalent amount of empty LNPs (LNP), or with vehicle (veh, water). Representative histograms show the percentage of CD40-, CD80-, CD86-, CCR7-, and MHC-II-positive cells assessed by flow cytometry, and IL-12 concentrations measured by ELISA ±SEM at 24 or 48 h post-treatment (n = 9 at 24 h; n = 3 at 48 h). ***$p < 0.001$ versus vehicle (two-way ANOVA, Dunnett's test).

However, a pronounced reduction in signal intensity was observed 24 h post-administration across all regions, with the most significant decreases in the thoracic and the abdominal areas for the unmod sequence, indicating a transient activation followed by downregulation of this pathway.

Taken together, these results reveal spatiotemporal activation of pro- and anti-inflammatory signaling pathways following systemic mRNA vaccination. Shortly after administration, the earliest and most prominent responses were observed in the abdominal area (predominantly containing the liver), for both NF-κB and STAT6 signaling. In this region, NF-κB activation was stronger and more sustained in response to unmodified mRNA, returning to baseline levels approximately 24 h after administration. In contrast, STAT6 activation was detected following both vaccines. Notably, at 24 h post-administration, STAT6 activity decreased below baseline levels, supporting the hypothesis of an active shutdown of STAT6-dependent transcription following an initial activation phase. Given that this reduction was observed across nearly all anatomical regions, it suggests a generalized systemic response. Overall, these data indicate that abdominal organs exhibit pronounced STAT6 and NF-κB modulation during the early phase of systemic mRNA vaccine administration.

Ex vivo assays to study organ-specific activation

To obtain a comprehensive view of the organ-specific activation profile, bioluminescence imaging was performed on dissected organs both 5 h after intravenous vaccine administration and at the end of the experiment (day 29) in reporter mice, following the experimental setup previously described. This analysis enabled the identification of organs primarily involved in STAT6 and NF-κB modulation. At the same time point, cytokine production profiles were assessed in plasma, to further characterize the immune responses elicited by the two vaccine formulations, while antigen-specific antibody titers were quantified on day 29. A cohort of vehicle-treated animals (glucose solution) was included to better delineate the contribution of the LNP component.

At 5 h post-administration (Figures5A and S4), NF-κB activation was observed in the thymus and liver of mice receiving the unmodified sequence, with a general upward trend across multiple organs. In contrast, mice treated with the modified sequence or with LNPs alone exhibited activation levels comparable to vehicle-treated controls, except for a mild activation detected in the liver of the mod-m1Ψ group. Regarding STAT6 activation, the unmodified sequence induced a high level of activation in the liver, whereas LNP and mod-m1Ψ elicited comparable bioluminescence signals, both of which were higher than those observed in vehicle-treated mice. In contrast, the mod-m1Ψ mRNA predominantly triggered activation in the thymus. Intramuscular administration of the unmod vaccine resulted in an activation pattern involving the same organs as systemic delivery, with the notable difference that a statistically significant activation was also observed in the inguinal lymph nodes and in the injected muscle exclusively in the NF-κB reporter (Figure S5).

Cytokine profiling in the plasma at 5 h after intravenous injection revealed upregulation of IL-12p70, CXCL10, and IFN-β in both vaccine groups relative to control and LNP-treated mice, whereas CCL2 and IFN-α were selectively increased in animals administered the unmodified mRNA, while no statistically significant modulation was observed for IL-1β, IL-10, INF-γ, and CXCL1 (Figures 5B and S6). These data indicate that both vaccines induced distinct immunostimulatory signatures primarily attributable to the mRNA chemistry rather than to the LNP formulation.

At day 29 (Figure 5C), bioluminescence imaging demonstrated sustained activation of NF-κB and STAT6 in the thymus of both vaccine groups. Moreover, STAT6 activation persisted in the liver and colon of mice treated with the modified sequence. The absence of non-specific upregulation of either NF-κB or STAT6 pathways in other organs suggests that the inflammatory response remained moderate and spatially confined at this time point. Plasma cytokine analysis revealed a moderate downregulation of CXCL1 compared with vehicle-treated mice across all treatments. In contrast, only LNP-treated mice showed a modest increase in IL-1β, GM-CSF, and

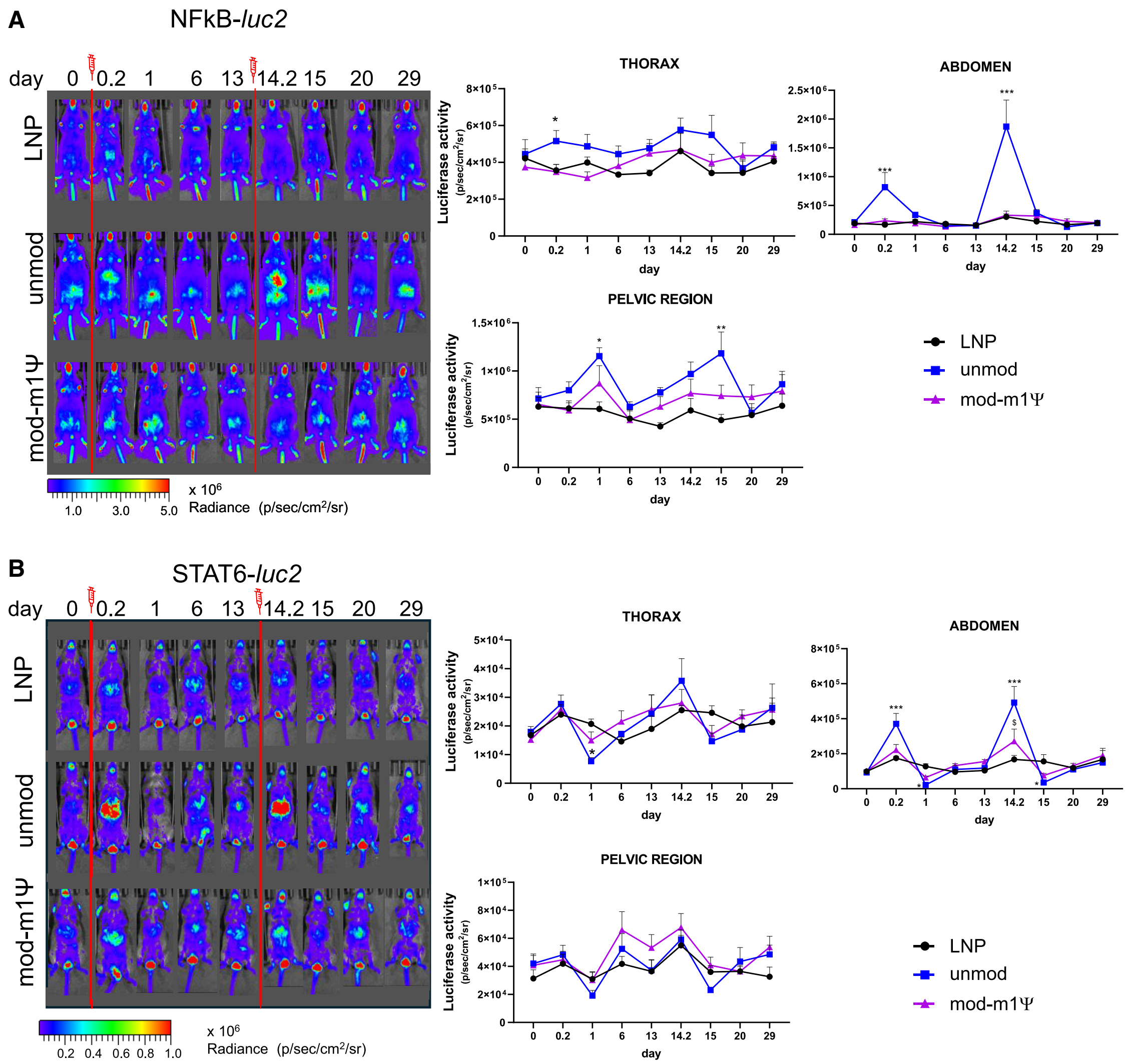

Figure 4. *In vivo* pro-inflammatory and anti-inflammatory mRNA vaccine responses in reporter mice

(A) NFκB-luc2 or (B) STAT6-*luc2* mice were injected intravenously (10 μg) with unmodified mRNA vaccine (unmod), modified mRNA vaccine (mod-m1Ψ), or the corresponding amount of empty LNPs (LNP) on days 0 and 14. Representative longitudinal *in vivo* bioluminescence images (0, 0.2, 1, 6, 13, 14.2, 15, 20, and 29 days) are shown on the left and displayed using pseudocolors according to the scale bar. Bioluminescence was quantified in selected regions of interest as defined in Figure 2. Bar graphs represent mean photon emission (photons/s/cm^2/sr) ± SEM (n = 6). *$p < 0.05$, **$p < 0.01$, ***$p < 0.001$ versus LNP (two-way ANOVA, Dunnett's test).

IFN-α, while no significant differences were observed for IFN-γ, CCL2, IL-10, IL-12p70, or IL-6 (Figures 5D and S6).

Finally, analysis of anti-antigen antibodies in plasma at day 29 showed that both vaccine formulations elicited detectable antigen-specific responses, with no significant differences in antibody titers between the two groups (Figure 5E).

Identification of important patterns of immunogenic potential

Given that the two vaccine formulations exhibited distinct NF-κB and STAT6 activation profiles while ultimately inducing comparable antibody responses, we hypothesized that correlating bioluminescence data with antibody production could help identify key time points, organs, and anatomical regions relevant for effective immunization. This approach aimed to define which organ-specific and temporal activation profiles of STAT6 and NF-κB are most conducive to antibody production.

Initially, we correlated bioluminescence intensity measured at day 29 in dissected organs (Figure 5C) from LNP-, modified-, and unmodified-treated mice with antigen-specific antibody levels (Figure 5E). As shown in Figure 6A, STAT6 activation exhibited a strong positive correlation with antibody titers in the thymus, colon and duodenum, whereas NF-κB activity did not display any consistent association with antibody production at any time point.

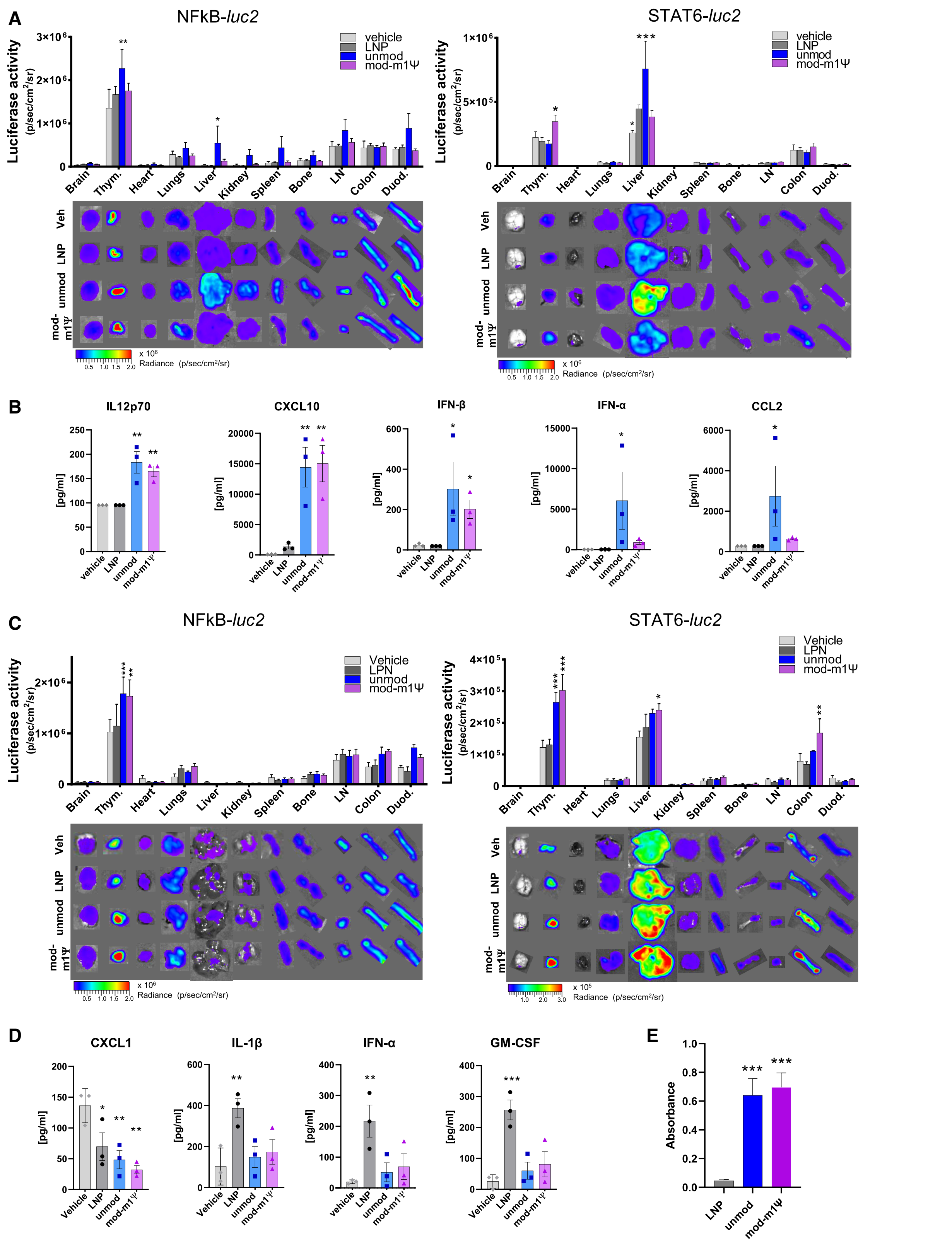

(legend on next page)

We next investigated whether the *in vivo* bioluminescence dynamics were associated with the capacity of the vaccine to induce an effective humoral response. To this end, bioluminescent signals detected in specific anatomical regions at different time points were correlated with plasma antibody levels measured at day 29. As shown in Figures 6B and 6C, several time points and anatomical regions displayed statistically significant correlations between bioluminescence intensity and antibody titers. Notably, the early STAT6-mediated response appeared to be the most predictive of immunogenic potential (Figure 6B). Specifically, abdominal bioluminescence at 5 h, day 13, and in the pelvic region at day 29 post-immunization exhibited strong positive correlations with antibody levels at day 29. Conversely, inverse correlations were observed for STAT6 signals detected at 1 day post-immunization in the abdominal and thoracic regions, as well as at day 15 in the thoracic area.

Regarding NF-κB activity, a positive correlation was observed in the thoracic region at day 13, whereas an inverse correlation was found in the abdominal region at day 6 (Figure 6C).

Together, these findings suggest that early STAT6 activation is more predictive of a successful immune response than NF-κB activation. In contrast, the subsequent attenuation of STAT6 activity in abdominal and thoracic organs appears to be required for optimal immunization. Moreover, the presence of distinct "waves" of STAT6- and NF-κB-mediated activation across different anatomical regions supports the notion of a temporally and spatially coordinated immune regulation, underscoring the complex interplay of these signaling pathways during vaccine-induced immunity.

DISCUSSION

In this study, we report the validation of a STAT6 reporter mouse capable of real-time, whole-body monitoring of STAT6-dependent immunomodulatory activity. This model enables both dynamic *in vivo* imaging and organ-specific *ex vivo* analyses. Efficient excision of the STOP cassette confirms the functionality of the flox system, indicating that crossing STAT6-STOP reporter mice with tissue-specific Cre drivers could facilitate compartment-restricted investigation of STAT6 biology. STAT6-*luc2* reporter mice faithfully detect the expected anti-inflammatory responses following administration of cytokines such as IL-4 and IL-13. Notably, the responses exhibit spatial and dose-dependent differences, with a prominent abdominal bioluminescence signal primarily originating from the liver—an organ characterized by high *Arg1* expression and well-established sensitivity to STAT6-mediated transcription.[29,34,35] These features establish the model as a powerful platform for *in vivo* investigation of STAT6 signaling. Notably, this system provides complementary insights to the pro-inflammatory responses captured by NFκB-*luc2* reporter mice.[21] When used in combination, these dual-reporter models offer a comprehensive and high-resolution physiological view of immune activation, representing a valuable toolkit for studying the spatial and temporal regulation of immunity *in vivo*.

Applying these reporter mice to LNP-encapsulated mRNA vaccination enabled a spatially and temporally resolved comparison of immune modulation between two systemically administered vaccine formulations encoding the same antigen but differing in RNA chemistry. As expected, unmodified mRNA elicited stronger early innate activation than its N^1-methylpseudouridine ($m^1\Psi$)-modified counterpart, reflected in heightened NF-κB signaling. This is consistent with the reduced TLR7/8 and RIG-I stimulation by $m^1\Psi$-containing transcripts and corroborated by plasma cytokine profiling at 5 h post-administration, which revealed induction of IFN-α and CCL2 specifically by unmodified mRNA. In fact, within hours of intravenous administration, NF-κB activation was pronounced in the liver and thymus; for the unmod vaccine, STAT6 activation was observed in the abdomen and liver, whereas the modified formulation induced STAT6 signaling primarily in the thymus.[22] Interestingly, LNPs alone were sufficient to trigger a STAT6 response at 5 h post-administration. Comparison of activation profiles following systemic and intramuscular administration of the unmodified vaccine at an early time point (5 h) revealed a largely overlapping organ distribution, with the notable exception of the draining lymph nodes and the injected muscle, where activation was observed exclusively in the NF-κB reporter. These findings are consistent with previous preclinical studies and regulatory assessments indicating that LNPs administered via intramuscular injection reach the draining lymph nodes but can also be detected in the liver across multiple species, including mice, rats, and non-human primates.[36–38] In line with these observations, repeat-dose toxicity studies for Comirnaty have reported transient histopathological changes at the injection site, in lymph nodes, and in the liver of treated animals.[38]

Our findings align with the fact that the liver is a key organ for safety monitoring in LNP-based vaccines, particularly for formulations with hepatic tropism. In this context, reporter models can support the rational design and preclinical evaluation of mRNA-LNP vaccines by linking tissue tropism with pathway-specific activation, thereby improving candidate selection and informing biodistribution and toxicology studies.

Figure 5. *Ex vivo* bioluminescence analysis, plasma cytokine profiling, and antigen-specific antibody responses

(A–C) Representative *ex vivo* bioluminescence images of organs dissected from NFκB-*luc2* and STAT6-*luc2* reporter mice at (A) 5 h or (C) 29 days after treatment. Signals are shown using pseudocolors according to the scale bar. Quantification of organ bioluminescence is shown in the bar graphs, representing mean photon emission (photons/s/cm^2/sr) ± SEM (n = 6). *$p < 0.05$, **$p < 0.01$, ***$p < 0.001$ versus LNP (two-way ANOVA, Dunnett's test). Thym, thymus, LN, right and left inguinal lymph nodes, Duod., duodenum. (B–D) Cytokine levels in plasma were measured (B) 5 h after administration or (D) at day 29. Cytokine concentrations are presented as bar graphs ±SEM (n = 3). *$p < 0.05$, **$p < 0.01$ versus vehicle (one-way ANOVA, Dunnett's test). (E) Antigen-specific antibodies were measured in plasma at day 29 by ELISA. Bar graphs represent mean absorbance ±SEM (n = 12). ***$p < 0.001$ versus LNP (two-way ANOVA, Dunnett's test).

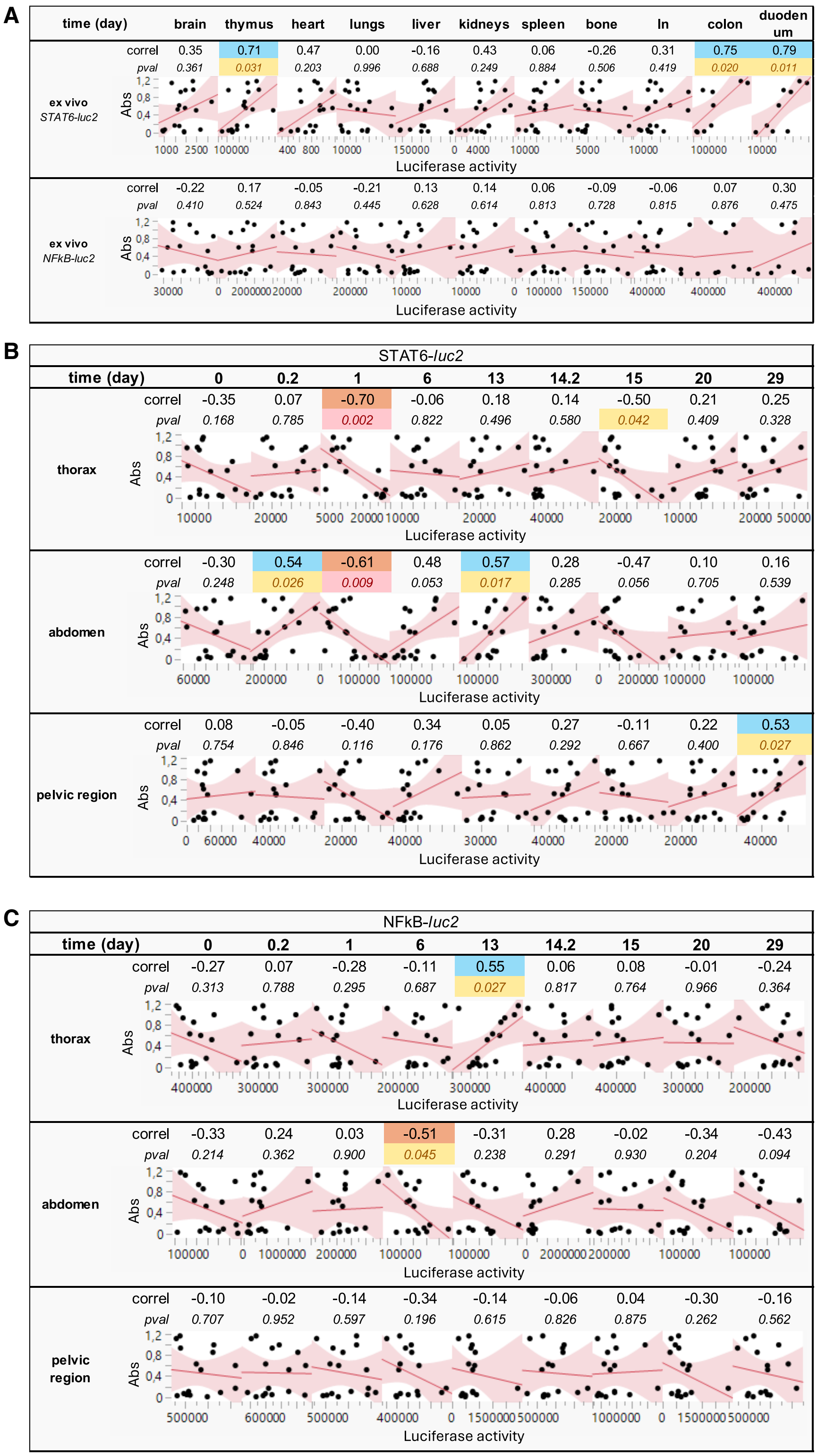

Figure 6. Correlations between antigen-specific antibodies and bioluminescence signals

(A) Correlations between *ex vivo* bioluminescence of dissected organs at day 29 (abscissa, luciferase activity [photons/s/cm^2/sr]) and antigen-specific antibody absorbance (ordinate, Abs) values are shown, with Pearson's correlation coefficients (r; correl) and *p* values (pval) for each interpolated curve. ln, lymph nodes. (B–C) Correlation analyses between *in vivo* bioluminescence signals in selected regions at defined time points (abscissa, luciferase activity [photons/s/cm^2/sr]) and antigen-specific antibody absorbance in plasma at day 29 (ordinate, Abs) are shown for (B) *STAT6-luc2* mice and (C) *NFκB-luc2* mice. Pearson's r (correl) and corresponding *p* values (pval) are reported for each interpolated curve.

Strategically located at the confluence of arterial and venous blood, the liver continuously monitors both systemic and gut-derived molecules, functioning as a central immunological hub that integrates environmental cues to maintain a balance between tolerance and immunity. It contains a dense network of immune cells—including Kupffer cells (80%–90% of all tissue macrophages), DCs, and natural killer cells—expressing diverse immune receptors.[39] Under steady-state conditions, the liver promotes tolerogenic responses while retaining the capacity to mount effective adaptive immunity when required.[39] In non-inflammatory settings, such as exposure to gut-derived LPS in the absence of danger signals, the liver maintains a tolerogenic environment characterized by low expression of MHC and co-stimulatory molecules and the presence of inhibitory signals. In contrast, during infection or inflammation, or upon exposure to additional pathogen-associated molecular patterns (e.g., CpG DNA), this tolerogenic state can be overridden. These conditions drive the upregulation of MHC and co-stimulatory molecules on Kupffer cells and other antigen-presenting cells, alongside a reduction in inhibitory signaling, thereby enabling efficient $CD4^+$ T cell activation and supporting $CD8^+$ T cell proliferation and differentiation into functional effector cells.[39] In our experimental setting, the liver displays a rapid and robust response, underscoring its potential role in shaping the early phases of immune activation. Only a few studies have examined the immunological impact of LNP-based vaccines on the liver; in this context, our observations align with those of Pongma et al.,[40] who demonstrated that, 7 days after mRNA/LNP vaccination, liver-associated macrophages undergo changes in gene expression and epigenetic profiles, highlighting the liver's active participation in immune responses rather than its role as a passive nanoparticle depot. These findings should be interpreted within a dynamic framework in which tolerogenic and immunogenic functions coexist and are modulated by the nature of the stimulus. Accordingly, the observed balance between pro- and anti-inflammatory signals is consistent with the liver's role as an immunological sentinel. However, the mechanisms governing the magnitude, duration, and triggers of this activation remain to be fully elucidated.

By 24 h, a subsequent wave of NF-κB activation emerged in the pelvic region for both vaccines, associated with a systemic reduction in STAT6 signaling. The timing is consistent with the 6- to 24-h window required for DC migration and initiation of antigen presentation, and this pattern likely reflects activation of lymph nodes during the onset of T cell priming.[11,12,41] The reduction of STAT6 signaling by 24 h is consistent with its well-described transient kinetics. Early responses to vaccination are dominated by Th1-polarizing cytokines and type I interferons, which inhibit STAT6 phosphorylation, suppress IL-4 production, and limit Th2 differentiation.[2–4] Prolonged STAT6 activity would risk undesirable Th2 polarization, IgE class switching, and pro-fibrotic gene expression; therefore, the reporter probably highlights the feedback mechanisms actively terminate STAT6 signaling.[42,43] The absence of sustained STAT6 activity is therefore mechanistically consistent with effective immunization and likely supports immunological protection.

Notably, at 29 days, NF-κB and STAT6 activity persisted in the thymus for both vaccines, consistent with ongoing processes such as thymocyte proliferation and the balance between maturation and apoptosis.[2–4] Interestingly, although evidence on thymic involvement following mRNA vaccination remains limited, increased metabolic activity in the thymus and axillary lymph nodes has been reported in children and young adults after COVID-19 mRNA vaccination, as assessed *in vivo* by ^{18}F-fluorodeoxyglucose uptake using PET imaging.[44,45] Such signals were detectable from several days up to months after immunization, suggesting that the thymus may contribute to immune responses beyond the early phases of vaccination. Given the well-established age-related thymic involution, the magnitude and nature of thymic modulation may differ in aged mice, potentially reflecting the altered immune responsiveness observed in elderly individuals in real-world settings.[46] In this context, these reporter models may provide a valuable platform to investigate potential associations between differential STAT6 and NF-κB activation and age-dependent immune responses, while also refining the interpretation of PET imaging findings.

In contrast, the lack of detectable changes in luciferase expression in lymph nodes and spleen at 29 days, where mechanistic effects might be expected, may reflect differences in the kinetics or type of pathway activation, vaccine-specific effects, or the route of administration, as well as limited statistical power due to low signal intensity, thereby necessitating larger sample sizes. Interestingly, for the modified vaccine, STAT6 activation was also observed at 29 days in the liver and colon, suggesting organ-specific long-term immune modulation beyond the thymus. The lack of systemic NF-κB activation is consistent with the absence of upregulation of pro-inflammatory plasma cytokines at 29 days, suggesting that these vaccines do not elicit prolonged inflammatory responses that could be potentially detrimental.

Integration of imaging bioluminescent data with antibody measurements revealed that early pathway dynamics were predictive of immunogenicity. Among all time points, the most informative and predictive was the early (5 h) STAT6 activation in the abdomen, followed by rapid deregulation in the abdomen and thorax by 24 h, a signal that correlates with antibody responses. This identifies STAT6 as a useful early biomarker of effective antibody production. In contrast, strong early NF-κB activation did not correlate with immunogenic antibody production, consistent with previous reports suggesting that excessive inflammation may compromise optimal adaptive responses.[47] In line with this, since unmodified mRNA induces stronger, potentially detrimental inflammation without improving antibody responses, it may not represent an optimal solution in our experimental setting.

Together, these findings demonstrate that NF-κB and STAT6 reporter mice provide a high-resolution platform for assessing vaccine performance and dissecting early immunoregulatory pathways. Because predictive signatures emerge within the first 24 h, this strategy could be further developed to enable rapid *in viv*o screening of

candidate vaccines, allowing prioritization of leads that recapitulate the transcription factor dynamics of highly immunogenic vaccines, without waiting for antibody readouts, and thereby accelerating vaccine development.

Study limitations

This study was performed in mice aged 3–6 months, an age range commonly considered to approximate young adulthood in humans. Given the strong age dependence of immune responses, including the effects of immunosenescence and inflammaging on adaptive immunity and vaccine responsiveness, these findings should be interpreted within the context of young adult immune competence.[46]

It should also be noted that systemic routes of administration, although not typically preferred for antiviral mRNA vaccines, are actively explored in the context of cancer vaccines.[48] In the present study, this approach was adopted to achieve controlled systemic biodistribution. However, alternative routes of administration, as well as formulations with distinct tissue tropisms or dosing regimens, may result in different activation profiles.

Furthermore, vaccine efficacy can ultimately be assessed only in appropriate disease models, as intrinsic factors such as vaccine design, delivery system, and disease context critically influence protective outcomes. Accordingly, the signaling biomarkers identified here should be interpreted within a dynamic framework in which additional factors—such as mRNA stability, translation efficiency, antigen persistence, and biological variability between animals—may contribute to differences in final antibody levels. Therefore, these reporter models are not intended as standalone tools for evaluating vaccine efficacy, but rather as an initial platform for high-throughput screening to identify vaccine configurations with optimal immune profiles and to inform targeted pharmacokinetic and safety studies, as well as to support basic research.

Conclusions

In summary, we present and validate complementary STAT6 and NF-κB reporter mice that enable real-time, organ-resolved monitoring of key regulatory pathways shaping early immune responses. Using these tools, we demonstrate that LNP-based mRNA vaccination elicits highly coordinated, yet chemistry-dependent, immune dynamics. This enables a detailed analysis of the spatiotemporal patterns of STAT6 and NF-κB signaling, with the liver emerging as an early hub for both pathways. These findings suggest that hepatic immune crosstalk should be studied in detail, as it may represent a critical determinant of downstream adaptive immunity during LNP-mediated vaccination, while also representing a key organ for pharmacological safety assessment. In addition, our data identify transient early STAT6 activation, rather than strong NF-κB-driven inflammation, as a positive predictor of antibody responses, highlighting the importance of balanced innate signaling in effective immunization. These insights support the use of STAT6 and NF-κB reporter mice as a tool for basic mechanistic research as well as to develop rapid *in vivo* screening approaches for the early identification of promising LNP-vaccine formulations.

MATERIALS AND METHODS

Plasmid construction

The plasmid *p2xSTAT6-GFP-luc2* was generated by inserting the promoter region from *p2xSTAT6-Luc2P* (a gift from Axel Nohturfft; Addgene plasmid #35486, RRID:Addgene 35486) upstream of the *loxP-STOP-loxP-GFP-FRT-IRES-luc2-FRT-polyA* cassette using the *XhoI* and *MluI* restriction sites. The floxed STOP cassette was then excised using Cre recombinase (New England Biolabs, M0298S) according to the manufacturer's protocol prior to performing cellular transfection assays. The FRT sites could be used to excise *luc2* through FLP recombinase. To generate the targeting vector, the resulting *2xSTAT6-loxP-STOP-loxP-GFP-flox-IRES-luc2-flox* cassette was cloned into the targeting backbone via site-directed recombination (VectorBuilder). In the final construct, the transgene was flanked by MARs and by homologous arms corresponding to chromosome 7, locus 21.[21]

Generation of STAT6-STOP and STAT6-*luc2* reporter mice

The targeting vector was linearized with *NotI* and transferred into 129/Sv embryonic stem cells by electroporation, using 35 μg of DNA for each 15 million cells (Core Facility for Conditional Mutagenesis, DIBIT San Raffaele). Positive clones were selected with puromycin (1 μg/mL), and one hundred resistant clones were screened for homologous recombination by PCR. A positive clone was injected into C57BL/6NCrL blastocysts and transferred to pseudopregnant CD1 females. The resulting chimeric male mice, with approximately 80%–90% chimerism, were bred with wild-type C57BL/6 J female mice to produce F1 transgenic mice named STAT6-STOP. This line was then crossed with B6.C-Tg(CMV-cre) 1Cgn/J mice to obtain STAT6-*luc2* reporter mice.

Immortalized cell cultures and transient transfections

RAW 264.7 and HT-29 cell lines were obtained from the American Type Culture Collection (ATCC) and cultured in Dulbecco's modified Eagle medium (DMEM; Gibco, Cat. 11965–092) supplemented with 10% fetal bovine serum (FBS; Gibco, Cat. A5209502), 1 mM Sodium Pyruvate (Gibco, Cat.11360070) and 1% Antibiotic-Antimycotic (Gibco, Cat. 15240–062) and maintained at 37°C in a humidified atmosphere containing 5% CO_2. Both cell lines were transfected with *p2xSTAT6-GFP-luc2* plasmid. For transfection, 50,000 RAW 264.7 cells were seeded in a 24-well plate and 60,000 HT-29 cells were seeded in a 48-well plate, cultured overnight prior to transfection. Transfection of RAW 264.7 cells was performed using FuGENE HD Transfection Reagent (Promega, Cat. 2311) with a DNA:Fugene ratio of 2:6 (μg:μL) for each well; for HT-29 the transfection was performed using Lipofectamine LTX Transfection Reagent (Thermo Fisher Scientific, Cat. 15338100) with a DNA: Lipofectamine ratio of 1:4.8 (μg:μL) following the manufacturer's instructions. HT-29 cells were co-transfected with *the pCDH-EF1-Luc2-P2A-tdTomato* (a gift from Kazuhiro Oka, Addgene plasmid #72486, 10% of total DNA). Transfected cell lines were treated

with IL-4, IL-13, IL-10 (PeproTech, Cat. 200–04, 200–13, 200–10, respectively), for 24 h or LPS (Merck, Cat. L2630) for 6 h or 24 h. Fluorescence images of live transiently transfected cells were acquired using an Axiovert 200 M microscope with dedicated software (AxioVision Rel 4.9, Zeiss) at a magnification of ×20.

Bone-marrow-derived and peritoneal macrophage isolation and culture

Mouse peritoneal macrophages were isolated by peritoneal lavage using 5 mL of pre-chilled 0.9% NaCl injected into the peritoneal cavity with a 21G needle. Recovered cells were centrifuged at 1,500 rpm for 5 min and subjected to red blood cell lysis with ACK buffer (0.15 M NH_4Cl, 1 mM $KHCO_3$, 0.1 mM EDTA; pH 7.3) for 5 min at 4°C. Cells were then seeded at 1×10^6 cells/well in 24-well plates in RPMI+GlutaMAX (Thermo Fisher Scientific, Cat. 61870036) supplemented with 10% endotoxin-free FBS (Gibco, Cat. A5209502), 1% penicillin/streptomycin (Gibco, Cat. 15240–062), and sodium pyruvate (Gibco, Cat. 11360070). After 45 min, non-adherent cells were removed by extensive PBS washing, and adherent macrophages were maintained in RPMI containing 10% FBS. Treatments were performed the following day.

Bone-marrow-derived macrophages (BMDMs) were generated by flushing bone marrow from tibiae and femora with DMEM + GlutaMAX (Gibco, Cat. 11965–092) using a 21G needle. Cells were centrifuged at 1,250 rpm for 8 min, seeded in T75 flasks in DMEM + GlutaMAX supplemented with 10% endotoxin-free FBS, 1% penicillin/streptomycin, and 1% sodium pyruvate, and incubated overnight. The following day, cells were collected, centrifuged, and reseeded at 3×10^6 cells/dish for differentiation over 6 days in DMEM + GlutaMAX containing 20% endotoxin-free FBS, 30% L929 cell-conditioned medium (ATCC), 1% penicillin/streptomycin, and 1% sodium pyruvate. After differentiation, BMDMs were detached using Accutase (Merck-Millipore, Cat. SCR005) and plated in 24-well plates at 4.5×10^5 cells/well. After 45 min, cells were extensively washed with PBS and maintained in DMEM containing 10% FBS. Treatments were performed the following day.

Animal treatments and *in vivo* and *ex vivo* imaging

All animal experimentation was carried out in accordance with the Animal Research: Reporting of In Vivo Experiments (ARRIVE) guidelines and European guidelines for animal care. Animal study protocols were approved by the Italian Ministry of Research and University (permission numbers: 417/2018, 416/2022, 904/2023, 105/2024). Experiments conducted in Finland complied with the Finnish Act on the Protection of Animals Used for Scientific or Educational Purposes (497/2013) and the Directive 2010/63/EU of the European Parliament and of the Council, and they were approved by the Finnish National Animal Experiment Board (Hankelupalautakunta – ELLA). Male mice of 3–6 months were used unless otherwise specified. The animals were provided *ad libitum* access to food and housed in individually ventilated plastic cages at a temperature range of 22°C–25°C with a relative humidity of 50% ± 10%. The housing environment followed an automatic cycle of 12 h of light and 12 h of darkness. To minimize any circadian influence, both the treated and control groups were analyzed at the same time, and the reference point (t0) for the start of the experiments was set in the morning (between 9:00 and 10:00 a.m.). For *in vivo* validation, an intraperitoneal injection of IL-4, IL-13 (PeproTech, Cat. 214–14 and 210–13) or LPS (Merck, Cat. L2630) was performed in a saline-based solution. For the vaccine experiment a tail vein injection was performed with 10 μg of mRNA or the corresponding amount of empty LNPs or vehicle (glucose-based solution). For Figure S5 the injection was performed in the leg muscle. For semi-quantitative bioluminescent photon-emission analysis, we followed established protocols.[49] Mice received a subcutaneous injection of luciferin (Promega, Cat. P1041; 80 mg/kg) 10 min prior to imaging. Animals were anesthetized with isoflurane and maintained under anesthesia throughout each 2-min optical imaging session. Bioluminescent imaging was performed using a charge-coupled device system (IVIS SpectrumCT, PerkinElmer). Following the final *in vivo* acquisition, mice were euthanized via cervical dislocation. Organs were immediately subjected to a 2-min *ex vivo* imaging session and then rapidly frozen at −80°C for subsequent analyses. Photon emission in defined regions was quantified using Living Image Software v4.2 (PerkinElmer). For biodistribution analyses with the fluorescent vaccine, mice were acquired using IVIS SpectrumCT (PerkinElmer), at different time points: after 2, 10, 30 min, and 6, 24, 48 h after tail vein injection of the fluorescent vaccine. At the end of the experiments, images were scaled and analyzed by applying the same regions of interest, using the Living Image 4.7.4 Software (PerkinElmer). Fluorescent signals were expressed as average radiance efficiency ([photons/second/square centimeter/steradian]/[μW/square centimeter]) which is a calibrated measurement of photon emission.

Vaccine production: Plasmid DNA design, expansion, and template linearization

Plasmid DNA for *in vitro* transcription (IVT) was designed to include an optimized T7 promoter compatible with CleanCap AG (3′OMe) co-transcriptional capping, a human HBB-derived 5′ UTR (5′ UTR), and a coding sequence (CDS) consisting of a mouse Ig κ-chain signal peptide fused in-frame to the antigen of interest. The 3′ region of the construct contained two tandem copies of the human HBB-derived 3′ UTR (HBB2x), followed by a 100-nt poly (A) tail positioned immediately upstream of a unique *HindIII* restriction site within the pUC57 backbone to enable template linearization. The plasmid was synthesized by GenScript and sequence verified. The plasmid was transformed into *Escherichia coli Stbl3* chemically competent cells (Invitrogen, Cat. C737303) by heat-shock transformation. Briefly, plasmid DNA was added to competent cells and incubated on ice for 30 min, followed by heat shock at 42°C for 45 s and immediate transfer to ice for 5 min. Pre-warmed Luria-Bertani (LB) broth was added, and cells were recovered by shaking at 37°C for 1 h before plating on LB agar plates containing ampicillin (100 μg/mL). Plates were incubated overnight at 37°C. Single colonies were inoculated into LB broth supplemented with ampicillin (100 μg/mL) and grown with shaking at 37°C for plasmid expansion.

Plasmid DNA was isolated using a Macherey-Nagel NucleoBond Xtra Maxi kit (Cat. 740424.10) according to the manufacturer's instructions. Purified plasmid DNA was quantified spectrophotometrically using a NanoPhotometer NP80 (Implen).

For IVT template preparation, plasmid DNA was linearized by digestion with *HindIII* (New England Biolabs, Cat. R3104L) at a ratio of 5 U enzyme per μg of DNA. Digestions were performed using plasmid DNA at a concentration of 100 ng/μL and incubated for 1.5 h at 37°C. Linearized DNA was purified by phenol-chloroform-isoamyl alcohol extraction (saturated with 10 mM Tris-HCl, pH 8.0, and 1 mM EDTA; Merck, Cat. P3803) by adding an equal volume of organic solvent, mixing thoroughly, and centrifuging at 14,000 × *g* for 10 min to separate phases. The aqueous phase was recovered, and DNA was precipitated by addition of 1/10 volume of 3 M sodium acetate (pH 5.2) and isopropanol, followed by overnight incubation at −20°C. DNA pellets were washed with 70% ethanol, air-dried, and resuspended in nuclease-free water. DNA concentration was re-quantified, and complete linearization was confirmed by agarose gel electrophoresis on a 1% agarose gel run at 100 V using a 1-kb DNA ladder (New England Biolabs, Cat. N3232S).

Vaccine production: *In vitro* transcription

Unmodified mRNA, N^1-methyl-pseudouridine-modified mRNA ($m^1\Psi$-UTP), and Cy5.5-labeled $m^1\Psi$-modified mRNA were synthesized by IVT using the MEGAscript T7 kit (Invitrogen, Cat. AMB13345), following the manufacturer's instructions. IVT reactions were performed using linearized plasmid DNA templates at a final concentration of 50 ng/μL. For unmodified transcripts, reactions contained standard ribonucleotide triphosphates (7.5 mM each) and CleanCap AG (3′-O-Me) (TriLink BioTechnologies, Cat. *N*-7413) at 5 mM. For modified mRNA, UTP was replaced with N^1-methyl-pseudouridine triphosphate ($m^1\Psi$-UTP; TriLink BioTechnologies). Fluorescently labeled mRNA was generated by supplementing the nucleotide mix with Cy5-UTP (MedChemExpress, Cat. HY-150145) at a 1:5 Cy5-UTP:UTP ratio. All IVT reactions were incubated at 37°C for 3 h and subsequently treated with TURBO DNase for 15 min at 37°C to remove residual plasmid DNA. mRNAs were then purified by LiCl precipitation by adding 7.5 M LiCl to a final concentration of 2.5 M, followed by overnight incubation at −20°C. RNA pellets were recovered by centrifugation at 14,000 × *g* for 20 min, washed once with 70% ethanol, briefly air-dried, and resuspended in nuclease-free water. RNA concentration and purity were measured using a NanoPhotometer NP80 (Implen), and transcript integrity was assessed by electrophoresis on a 1% agarose gel in 1× TAE buffer, run at 90 V for 1 h with an RNA ladder (New England Biolabs, Cat. N0362S) (Figure S7).

Vaccine production: Microfluidic mRNA-LNP formulation

mRNA-LNPs were formulated using the Sunshine microfluidic system (Unchained Labs) equipped with a 190XT mixer. Lipids were dissolved in ethanol at molar ratios of ALC-0315 (47.4%), cholesterol (40.9%), DSPC (10%), and ALC-0159 (1.7%); all lipids and solvents were obtained from Merck. The aqueous phase consisted of mRNA diluted to 0.16 mg/mL in 50 mM citrate buffer (pH 4). Organic and aqueous streams were co-injected into the microfluidic mixer at a total flow rate of 10 mL/min with a 1:3 ethanol-to-aqueous flow-rate ratio, yielding nanoparticles with an N/P ratio of 6 and a lipid-to-mRNA mass ratio of 25:1. Control empty LNPs (LNP) were formulated as described above, using an aqueous phase that did not contain mRNA. The resulting LNP suspensions were collected in RNase-free tubes and dialyzed using Slide-A-Lyzer dialysis cassettes (3.5 kDa MWCO; Thermo Scientific, Cat. 66330) against sucrose-phosphate buffer (150 mM sucrose, 75 mM NaCl, 10 mM sodium phosphate, pH 7.4), with buffer exchanges at 1 h, 2 h, and overnight. Final formulations were concentrated to 0.2 mg/mL using 100-kDa PES centrifugal filters (Sartorius Vivaspin, Cat. VS04T42), sterile-filtered through 0.22-μm PES membranes (Nalgene Cat. 720–1320), aliquoted, and stored at −80°C until use.

Vaccine production: Characterization of mRNA-LNPs

mRNA-LNPs were characterized after a single freeze-thaw cycle (Figure S7). Encapsulation efficiency was measured using the RiboGreen assay (Invitrogen, Cat. R11491). LNP formulations (0.2 mg/mL mRNA) were diluted 1:50 in 1× Tris-EDTA (TE) buffer prepared from TE 20× stock (Invitrogen, Cat. T11493), and 50 μL was plated into wells containing either 50 μL of 1× TE buffer or Triton buffer (0.2% Triton X-100 prepared in 1× TE). After a 10-min incubation at 37°C, an equal volume (100 μL) of RiboGreen reagent diluted 1:100 in 1× TE was added, and fluorescence was recorded at 485/535 nm. Encapsulation efficiency (EE) was calculated using fluorescence values obtained from samples in 1× TE buffer (free mRNA, F_free) and Triton buffer (total mRNA, F_total) according to the equation $EE (\%) = [1 - (F\_free/F\_total)] \times 100$, and it typically ranged from 89 to 96%. RNA content was interpolated from a standard curve (0.1–1.5 μg/mL) using the linear relationship $F = A \times C + B$ and corrected for the 200-fold dilution. Particle size and polydispersity index were determined by dynamic light scattering using a Malvern Panalytical Zetasizer after diluting 50 μL of LNPs into 950 μL of storage buffer. Measurements were performed using a solvent refractive index of 1.338 and viscosity of 1.0547 mPa · s, with LNP optical parameters set to a refractive index of 1.46, and absorption of 0.01. Zeta potential was determined by electrophoretic light scattering after dilution of LNPs in 0.1× sucrose-phosphate buffer, using solvent parameters of refractive index 1.331, viscosity 0.8884 mPa · s, and dielectric constant 78.5.

Luciferase assay

Luciferase assays were performed as previously described.[50] For cell-based assays, cells were lysed using 1× Luciferase Cell Culture Lysis Reagent (Promega, Cat. E1531) prior to biochemical analysis. For tissue assays, one-half of each organ was homogenized in lysis buffer and subjected to repeated freeze-thaw cycles, followed by centrifugation at 13,000 × *g* for 30 min to obtain the soluble protein fraction. Protein concentration was determined using the Bradford assay. Luciferase activity was measured in luciferase assay buffer, and relative luminescence units (RLU) were quantified in a 96-well plate

using a Veritas luminometer (Turner Biosystems) with a 10-s integration time. Luminescence values were normalized to total protein content as determined by the Bradford assay.

Reverse transcription quantitative PCR

Cells were lysed in TRIzol reagent (Thermo Fisher Scientific, Cat. 15596026), and total RNA was extracted from the aqueous phase using the Direct-zol RNA Miniprep Kit (Zymo Research, Cat. R2050) according to the manufacturer's instructions. RT-qPCR analyses were performed as previously described.[18] Briefly, cDNA was synthesized using Moloney murine leukemia virus reverse transcriptase (Promega, Cat. M3681) and random primers (Promega, Cat. C118A). For each sample, negative control reactions were prepared in the absence of reverse transcriptase.

RT-qPCR was performed using SYBR Green chemistry (Promega, Cat. A600150), and cDNA samples were amplified in triplicate in 96-well plates using GoTaq qPCR Master Mix (Promega, Cat. A6001) in a final reaction volume of 10 μL, according to the manufacturer's protocol, on a QuantStudio 3 96-Well 0.1 mL Block system (Thermo Fisher Scientific). The thermal cycling conditions were as follows: initial denaturation at 95°C for 2 min, followed by 40 cycles of 15 s at 95°C and 1 min at 60°C.

The primers used (Eurofins) were *Arg1* (5′-GAATCTGCATGGGCAACCT-3′; 5′-ACACGATGTCTTTGGCAGATAT-3′), *mChil3* (5′-TGGGCTAAGGACAGACCAAC-3′; 5′-ACTGAACGGGGCAGATCCAA-3′), and *Rplp0* (5′-GGCGACCTGGAAGTCCAACT-3′; 5′-CCATCAGCACCACAGCCTTC-3′). Gene expression levels were calculated using the comparative Ct method ($2^{-\Delta\Delta Ct}$), with Rplp0 used as the housekeeping gene.

Measurement of circulating cytokine levels

Plasma collected from treated mice was stored at −80°C until use. At the time of the experiment, plasma samples were thawed, diluted 2-fold in assay buffer, and cytokine levels were measured using the bead-based LEGENDplex Mouse Anti-Virus Response Panel (13-plex) immunoassay according to the manufacturer's instructions (Biolegend, Cat. 740622).

Generation and treatment of BM-DCs

BM-DCs were generated by culturing bone marrow cells for 8–10 days in DC medium consisting of RPMI (EuroClone, Cat. ECB9006L) supplemented with 10% fetal calf serum (Gibco, Cat. 10500–064), 10 ng/mL GM-CSF (PeproTech, Cat. AF-315-03), 100 U/mL penicillin (Orion, Cat. 465161), and 100 μg/mL streptomycin (Thermo Fisher Scientific, Cat. D7253), and 2 mM L-glutamine (Thermo Fisher Scientific, Cat. BP379–100). Cells were maintained at 37°C in a humidified atmosphere containing 5% CO_2. Medium was supplemented or refreshed on days 3, 6, and 8. On day 8, cells were replated and treated on day 9 for 24 or 48 h with 2.5 μg of mRNA formulated in LNPs or an equivalent amount of empty LNPs.

Flow cytometric analysis of BM-DC maturation

To assess the maturation status of BM-DCs, the following fluorochrome-conjugated antibodies were used: CCR7-PE, MHC-II-APC-eFluor780, CD80-APC (BioLegend, Cat. 120105, clone 4B12, 47-5321-82, clone M5/114.15.2, 17-0801-81, clone 16-10A1) and CD86-FITC (BD Biosciences, Cat. 11-0862-85, clone GL1). Fc receptor blocking (BD Pharmingen, Cat. 553142, clone 2.4G2) was applied in all experiments involving mouse cells. Unstained controls and fluorescence-minus-one controls were included for each panel. Data acquisition was performed on a BD LSRFortessa flow cytometer, and analysis was conducted using FlowJo software (Tree Star).

ELISA

The concentration of IL-12 in supernatants from BM-DCs was determined by ELISA using the Mouse IL-12/IL-23 p40 allele-specific DuoSet ELISA kit (R&D Systems, Cat. DY499) following the manufacturer's instructions. Briefly, 96-well MaxiSorp microplates (Thermo Fisher Scientific, Cat. 442404) were coated overnight with capture antibody. The following day, wells were washed and blocked with 1% bovine serum albumin (BSA) in PBS (Biowest, Cat. P6154; Lonza, Cat. 17-516 F) for at least 1 h. After washing, standards and samples were added and incubated for 2 h. Detection antibody was then applied for 2 h, followed by a 20-min incubation with Streptavidin-HRP (R&D, Cat. DY998). Substrate solution (R&D, Cat. DY499) was added until visible color developed (maximum 20 min), and the reaction was stopped with sulfuric acid (Acros Organics, Cat. 124645001, diluted 1:4 in water). Optical density was measured immediately at 450 nm, with 540 nm as the reference wavelength, using a Multiskan GO spectrophotometer (Thermo Fisher Scientific). IL-12 concentration was calculated from the standard curve. All washing steps were conducted with three washes of 0.05% Tween 20 in PBS (Fisher BioReagents, Cat. BP337), followed by one wash with PBS alone.

Antigen-specific antibodies in sera from immunized mice were quantified by ELISA. Briefly, recombinant goat antigen, kindly provided by Prof. Ricagno, was diluted to a final concentration of 10 μg/mL in 0.05 M carbonate coating buffer, and 100 μL per well was used to coat 96-well plates overnight at 4°C. Plates were blocked for 1 h at room temperature with PBS containing 1% (w/v) BSA (Sigma-Aldrich, Cat. 1120180025). Serum samples from immunized mice were diluted 1:100 in PBS containing 1% BSA, added to the wells (100 μL), and incubated for 2 h at room temperature. Plates were washed four times with PBS containing 0.1% Tween 20. Horseradish peroxidase-conjugated anti-mouse IgG secondary antibodies (Bio-Rad, Cat. 1706516) were diluted 1:5,000 in PBS containing 0.1% BSA, added to the wells, and incubated for 1 h at room temperature, followed by four washes with PBS containing 0.1% Tween 20. Signal detection was performed using 3,3′,5,5′-tetramethylbenzidine (TMB; Thermo Scientific, Cat. 34028). The reaction was stopped with 2 N sulfuric acid, and absorbance was measured using a GloMax Discover microplate reader (Promega).

Statistical analysis

Data are presented as the mean ± standard deviation unless otherwise noted in the figure legends. Statistical analyses were performed using Prism 7 (Version 8.00, GraphPad Software Inc.). Outliers were identified and excluded using the ROUT method with Q = 1. For comparisons between two groups, Student's *t* test was applied. One-way ANOVA was used to evaluate differences among three or more independent groups, followed by Tukey's post hoc test for pairwise comparisons or Dunnett's test for comparisons against a control group. Two-way ANOVA, followed by Sidak's post hoc test, was used to assess the effects of two factors in multiple comparisons. A p value <0.05 was considered statistically significant.

DATA AND CODE AVAILABILITY

The data that support the findings of this study are available from the senior author (paolo.ciana@unimi.it) upon reasonable request. The models are deposited at the Mutant Mouse Resource & Research Centers (MMRRC; www.mmrrc.org) NFkB-*luc2,* MMRRC: 076124-MU; NFkB-STOP, MMRRC: 076125-MU; STAT6-STOP, MMRRC: 076143-MU; STAT6-*luc2,* MMRRC: 076144-MU.

ACKNOWLEDGMENTS

The authors are grateful for financial support from PNRR M4C2-Investimento 1.4-CN00000041-PNRR_CN3RNA_SPOKE9 (to P.C.), from the Research Council of Finland and Finnish Cancer Foundation (to S.F.), and from the Department of Health Sciences, University of Milan, Linea 2, intramural funding (to E.B. and A.P.).

AUTHOR CONTRIBUTIONS

Conceptualization: E.B., N.R., A.V., C.S., S.P., C.D.V., L.O., D.M., S.F., S.M., E.V., P.C.; methodology: E.B., A.P., N.R., C.M., C.S., S.P., F.A., M.L.G., C.M., G.C., M.R., C.D.V., L. V., L.O.; investigation: E.B., A.P., N.R., C.M., C.S., S.P., F.A., M.L.G., C.M., G.C., M.R., C. D.V., L.V., L.O.; formal analysis: E.B., C.S., S.P., M.L.G.V., C.M., C.D.V., L.V., L.O.; funding acquisition: P.C., S.F.; supervision: L.O., D.M., D.F., S.M., E.V., P.C.; writing of the original draft: E.B., A.P., review and editing: L.O., D.M., D.F., S.M., E.V., P.C. All authors read and approved the final manuscript.

DECLARATION OF INTERESTS

The authors declare no competing interests.

SUPPLEMENTAL INFORMATION

Supplemental information can be found online at https://doi.org/10.1016/j.ymthe.2026.05.028.

REFERENCES

1. Chi, H., Pepper, M., and Thomas, P.G. (2024). Principles and therapeutic applications of adaptive immunity. Cell *187*, 2052–2078.

2. Kaech, S.M., and Cui, W. (2012). Transcriptional control of effector and memory CD8+ T cell differentiation. Nat. Rev. Immunol. *12*, 749–761.

3. Steinman, R.M. (2001). Dendritic cells and the control of immunity: enhancing the efficiency of antigen presentation. Mt. Sinai J. Med. *68*, 160–166.

4. Pulendran, B., and Ahmed, R. (2011). Immunological mechanisms of vaccination. Nat. Immunol. *12*, 509–517.

5. Papp, K.A., Haraoui, B., Kumar, D., Marshall, J.K., Bissonnette, R., Bitton, A., Bressler, B., Gooderham, M., Ho, V., Jamal, S., et al. (2019). Vaccination guidelines for patients with immune-mediated disorders on immunosuppressive therapies. J. Cutan. Med. Surg. *23*, 50–74.

6. Murphy, K., and Weaver, C. (2016). Janeway's immunobiology. Garland Science (New York: Taylor & Francis Group).

7. Kawai, T., and Akira, S. (2010). The role of pattern-recognition receptors in innate immunity: update on Toll-like receptors. Nat. Immunol. *11*, 373–384.

8. Turqueti-Neves, A., Otte, M., da Costa, O.P., Höpken, U.E., Lipp, M., Buch, T., and Voehringer, D. (2014). B-cell-intrinsic STAT 6 signaling controls germinal center formation. Eur. J. Immunol. *44*, 2130–2138.

9. Guldenpfennig, C., Teixeiro, E., and Daniels, M. (2023). NF-kB's contribution to B cell fate decisions. Front. Immunol. *14*, 1214095.

10. Rescigno, M., Martino, M., Sutherland, C.L., Gold, M.R., and Ricciardi-Castagnoli, P. (1998). Dendritic cell survival and maturation are regulated by different signaling pathways. J. Exp. Med. *188*, 2175–2180.

11. Sen, R., and Baltimore, D. (1986). Inducibility of k immunoglobulin enhancer-binding protein NF-kB by a posttranslational mechanism. Cell *47*, 921–928.

12. Oh, H., and Ghosh, S. (2013). NF-κB: roles and regulation in different CD 4+ T-cell subsets. Immunol. Rev. *252*, 41–51.

13. Tolomeo, M., and Cascio, A. (2024). STAT4 and STAT6, their role in cellular and humoral immunity and in diverse human diseases. Int. Rev. Immunol. *43*, 394–418.

14. Elo, L.L., Järvenpää, H., Tuomela, S., Raghav, S., Ahlfors, H., Laurila, K., Gupta, B., Lund, R.J., Tahvanainen, J., Hawkins, R.D., et al. (2010). Genome-wide profiling of interleukin-4 and STAT6 transcription factor regulation of human Th2 cell programming. Immunity *32*, 852–862.

15. Poli, M.C., Aksentijevich, I., Bousfiha, A.A., Cunningham-Rundles, C., Hambleton, S., Klein, C., Morio, T., Picard, C., Puel, A., Rezaei, N., et al. (2025). Human inborn errors of immunity: 2024 update on the classification from the International Union of Immunological Societies Expert Committee. J. Hum. Immun. *1*, e20250003.

16. O'shea, J.J., Lahesmaa, R., Vahedi, G., Laurence, A., and Kanno, Y. (2011). Genomic views of STAT function in CD4+ T helper cell differentiation. Nat. Rev. Immunol. *11*, 239–250.

17. Rizzi, N., Villa, A., Benedusi, V., Brunialti, E., Cesari, N., Ciana, P., and Maggi, A. (2018). Endocrine influence on neuroinflammation: the use of reporter systems. J. Neuroendocrinol. *30*, e12496. https://doi.org/10.1111/jne.12496.

18. Brunialti, E., Rizzi, N., Pinto-Costa, R., Villa, A., Panzeri, A., Meda, C., Rebecchi, M., Di Monte, D.A., and Ciana, P. (2024). Design and validation of a reporter mouse to study the dynamic regulation of TFEB and TFE3 activity through in vivo imaging techniques. Autophagy *20*, 1879–1894.

19. Ciana, P., Di Luccio, G., Belcredito, S., Pollio, G., Vegeto, E., Tatangelo, L., Tiveron, C., and Maggi, A. (2001). Engineering of a mouse for the in vivo profiling of estrogen receptor activity. Mol. Endocrinol. *15*, 1104–1113.

20. Maggi, A., Ottobrini, L., Biserni, A., Lucignani, G., and Ciana, P. (2004). Techniques: reporter mice–a new way to look at drug action. Trends Pharmacol. Sci. *25*, 337–342.

21. Rizzi, N., Rebecchi, M., Levandis, G., Ciana, P., and Maggi, A. (2017). Identification of novel loci for the generation of reporter mice. Nucleic Acids Res. *45*, e37.

22. Karikó, K., Muramatsu, H., Welsh, F.A., Ludwig, J., Kato, H., Akira, S., and Weissman, D. (2008). Incorporation of pseudouridine into mRNA yields superior nonimmunogenic vector with increased translational capacity and biological stability. Mol. Ther. *16*, 1833–1840.

23. Alexopoulou, L., Holt, A.C., Medzhitov, R., and Flavell, R.A. (2001). Recognition of double-stranded RNA and activation of NF-κB by Toll-like receptor 3. Nature *413*, 732–738.

24. Andries, O., Mc Cafferty, S., De Smedt, S.C., Weiss, R., Sanders, N.N., and Kitada, T. (2015). N1-methylpseudouridine-incorporated mRNA outperforms pseudouridine-incorporated mRNA by providing enhanced protein expression and reduced immunogenicity in mammalian cell lines and mice. J. Control Release *217*, 337–344.

25. Palmer-Crocker, R.L., Hughes, C.C., and Pober, J.S. (1996). IL-4 and IL-13 activate the JAK2 tyrosine kinase and Stat6 in cultured human vascular endothelial cells through a common pathway that does not involve the gamma c chain. J. Clin. Invest. *98*, 604–609.

26. O'Farrell, A., Liu, Y., Moore, K.W., and Mui, A.L.-. (1998). IL-10 inhibits macrophage activation and proliferation by distinct signaling mechanisms: evidence for Stat3-dependent and-independent pathways. EMBO J. *17*, 1006–1018.

27. Sun, F.-L., and Elgin, S.C.R. (1999). Putting boundaries on silence. Cell *99*, 459–462.

28. Schwenk, F., Baron, U., and Rajewsky, K. (1995). A cre-transgenic mouse strain for the ubiquitous deletion of loxP-flanked gene segments including deletion in germ cells. Nucleic Acids Res. *23*, 5080–5081.

29. Karlsson, M., Zhang, C., Méar, L., Zhong, W., Digre, A., Katona, B., Sjöstedt, E., Butler, L., Odeberg, J., Dusart, P., et al. (2021). A single–cell type transcriptomics map of human tissues. Sci. Adv. *7*, eabh2169.

30. Andrews, R.P., Ericksen, M.B., Cunningham, C.M., Daines, M.O., and Hershey, G.K. K. (2002). Analysis of the life cycle of stat6: continuous cycling of STAT6 is required for IL-4 signaling. J. Biol. Chem. *277*, 36563–36569.

31. Ishii, M., Wen, H., Corsa, C.A.S., Liu, T., Coelho, A.L., Allen, R.M., Carson, W.F., IV, Cavassani, K.A., Li, X., Lukacs, N.W., et al. (2009). Epigenetic regulation of the alternatively activated macrophage phenotype. Blood, J. Am. Blood *114*, 3244–3254.

32. Di, J., Du, Z., Wu, K., Jin, S., Wang, X., Li, T., and Xu, Y. (2022). Biodistribution and non-linear gene expression of mRNA LNPs affected by delivery route and particle size. Pharm. Res. *39*, 105–114.

33. Pardi, N., Tuyishime, S., Muramatsu, H., Kariko, K., Mui, B.L., Tam, Y.K., Madden, T.D., Hope, M.J., and Weissman, D. (2015). Expression kinetics of nucleoside-modified mRNA delivered in lipid nanoparticles to mice by various routes. J. Control Release *217*, 345–351.

34. Huttlin, E.L., Jedrychowski, M.P., Elias, J.E., Goswami, T., Rad, R., Beausoleil, S.A., Villén, J., Haas, W., Sowa, M.E., and Gygi, S.P. (2010). A tissue-specific atlas of mouse protein phosphorylation and expression. Cell *143*, 1174–1189.

35. Ricardo-Gonzalez, R.R., Red Eagle, A., Odegaard, J.I., Jouihan, H., Morel, C.R., Heredia, J.E., Mukundan, L., Wu, D., Locksley, R.M., and Chawla, A. (2010). IL-4/STAT6 immune axis regulates peripheral nutrient metabolism and insulin sensitivity. Proc. Natl. Acad. Sci. *107*, 22617–22622.

36. Zhang, X., Li, Y., and Zhou, Z. (2024). Lipid Nanoparticle-Based Delivery System─ A Competing Place for mRNA Vaccines. ACS omega *9*, 6219–6234.

37. Buckley, M., Araínga, M., Maiorino, L., Pires, I.S., Kim, B.J., Michaels, K.K., Dye, J., Qureshi, K., Zhang, Y.J., Mak, H., et al. (2025). Visualizing lipid nanoparticle trafficking for mRNA vaccine delivery in non-human primates. Mol. Ther. *33*, 1105–1117.

38. European Medicines Agency Assessment report, Comirnaty, Common Name: COVID-19 mRNA Vaccine (Nucleoside-modified), Procedure No. EMEA/H/C/005735/000. EMA/707383/2020.

39. Jenne, C.N., and Kubes, P. (2013). Immune surveillance by the liver. Nat. Immunol. *14*, 996–1006.

40. Pongma, C., Keawvilai, P., Boonmee, A., Wongprom, B., Pattarakankul, T., Sittplangkoon, C., Alameh, M.-G., Kueanjinda, P., Heyes, J., Lam, K., et al. (2025). Effect of mRNA formulated with lipid nanoparticles on the transcriptomic and epigenetic profiles of F4/80+ liver-associated macrophages. Sci. Rep. *15*, 1146.

41. Bonam, S.R., Hazell, N.C., Mathew, M.J., Liang, Y., Zhang, X., Wei, Z., Alameh, M.-G., Weissman, D., and Hu, H. (2024). Innate and adaptive immune parameters following mRNA vaccination in mice. Vaccines *12*, 543.

42. Dickensheets, H.L., Venkataraman, C., Schindler, U., and Donnelly, R.P. (1999). Interferons inhibit activation of STAT6 by interleukin 4 in human monocytes by inducing SOCS-1 gene expression. Proc. Natl. Acad. Sci. *96*, 10800–10805.

43. Knosp, C.A., and Johnston, J.A. (2012). Regulation of CD4+ T-cell polarization by suppressor of cytokine signalling proteins. Immunology *135*, 101–111.

44. von Tresckow, J., von Tresckow, B., Reinhardt, H.C., Herrmann, K., and Berliner, C. (2021). Thymic hyperplasia after mRNA based Covid-19 vaccination. Radiol. Case Rep. *16*, 3744–3745.

45. Luthria, G., Baratto, L., Adams, L., Morakote, W., and Daldrup-Link, H.E. (2024). Increased Metabolic Activity of the Thymus and Lymph Nodes in Pediatric Oncology Patients After Coronavirus Disease 2019 Vaccination. J. Nucl. Med. *65*, 22–24.

46. Nikolich-Žugich, J. (2018). The twilight of immunity: emerging concepts in aging of the immune system. Nat. Immunol. *19*, 10–19.

47. Alexandre, Y.O., Devi, S., Park, S.L., Mackay, L.K., Heath, W.R., and Mueller, S.N. (2020). Systemic inflammation suppresses lymphoid tissue remodeling and B cell immunity during concomitant local infection. Cell Rep. *33*, 108567.

48. Lorentzen, C.L., Haanen, J.B., Met, Ö., and Svane, I.M. (2022). Clinical advances and ongoing trials of mRNA vaccines for cancer treatment. Lancet Oncol. *23*, e450–e458.

49. Brunialti, E., Villa, A., Mekhaeil, M., Mornata, F., Vegeto, E., Maggi, A., Di Monte, D. A., and Ciana, P. (2021). Inhibition of microglial β-glucocerebrosidase hampers the microglia-mediated antioxidant and protective response in neurons. J. Neuroinflammation *18*, 22018.

50. Rizzi, N., Brunialti, E., Cerri, S., Cermisoni, G., Levandis, G., Cesari, N., Maggi, A., Blandini, F., and Ciana, P. (2018). In vivo imaging of early signs of dopaminergic neuronal death in an animal model of Parkinson's disease. Neurobiol. Dis. *114*, 74–84. https://doi.org/10.1016/j.nbd.2018.02.005.

YMTHE, Volume *34*

Supplemental Information

Spatiotemporal atlas of pro-inflammatory NF-κB and anti-inflammatory STAT6 signaling using reporter mice during mRNA vaccination

Electra Brunialti, Alessia Panzeri, Nicoletta Rizzi, Alessandro Villa, Clara Meda, Chiara Sfogliarini, Stefano Persano, Federico Arlati, Maria L. Guevara Lopez, Cristina Martelli, Gaetano Cannavale, Monica Rebecchi, Clara Di Vito, Letizia Venturini, Luisa Ottobrini, Domenico Mavilio, Susanna Fagerholm, Saverio Minucci, Elisabetta Vegeto, and Paolo Ciana

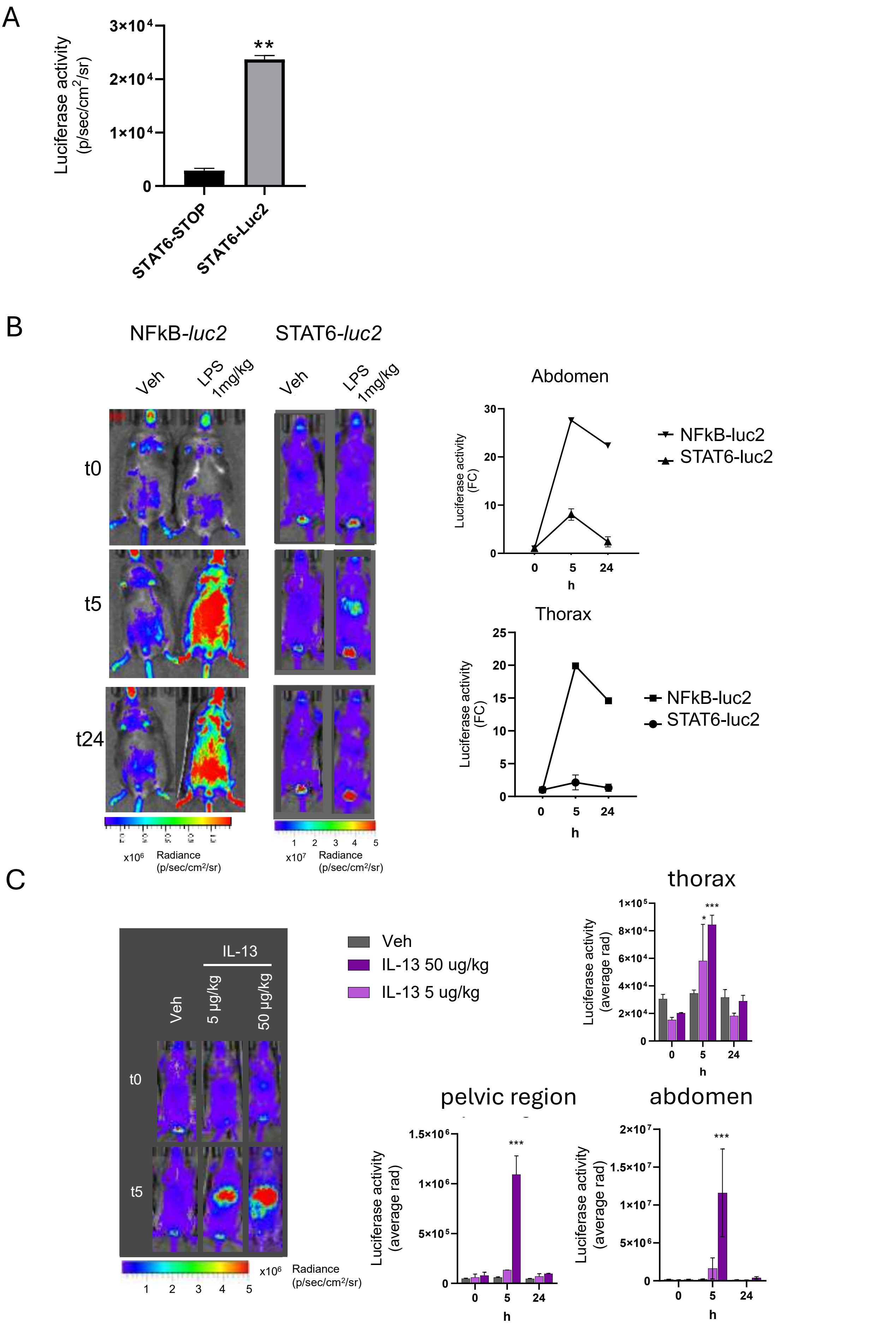
A
Luciferase activity (p/sec/cm²/sr)
3×10⁴
2×10⁴
1×10⁴
0
**
STAT6-STOP
STAT6-Luc2
B
NFkB-luc2
STAT6-luc2
Veh
LPS 1mg/kg
t0
t5
t24
x10⁶ Radiance (p/sec/cm²/sr)
1 2 3 4 5
x10⁷ Radiance (p/sec/cm²/sr)
Abdomen
Luciferase activity (FC)
30
20
10
0
0 5 24
h
NFkB-luc2
STAT6-luc2
Thorax
25
20
15
10
5
C
IL-13
Veh
5 µg/kg
50 µg/kg
x10⁶ Radiance (p/sec/cm²/sr)
Veh
IL-13 50 ug/kg
IL-13 5 ug/kg
thorax
Luciferase activity (average rad)
1×10⁵
8×10⁴
6×10⁴
4×10⁴
2×10⁴
*
***
pelvic region
1.5×10⁶
1×10⁶
5×10⁵
abdomen
2×10⁷
1.5×10⁷
1×10⁷
5×10⁶

Figure S1. STAT6 reporter mouse characterization. (A) Quantification of whole-body bioluminescence (BLI) in STAT6-STOP and STAT6-*luc2* mice. Data are presented as total luciferase activity (n = 2). Statistical significance was assessed using an unpaired *t*-test (**$p < 0.01$). **(B)** *in vivo* BLI of NFκB-*luc2 and* STAT6-*luc2* mice treated with LPS (1 mg/kg, intraperitoneally) at 0, 5, and 24 hours post-injection. Representative images (left) and corresponding quantification (right) are shown. Luciferase activity is expressed as fold change relative to baseline (t0). **(C)** In vivo bioluminescence imaging of STAT6-*luc2* mice following intraperitoneal administration of IL-13 (5 or 50 µg/kg). BLI was quantified in defined regions of interest (Figure 2) and is shown together with representative images. Statistical analysis was performed using two-way ANOVA followed by Dunnett's test versus vehicle (*$p < 0.05$, ***$p < 0.001$).

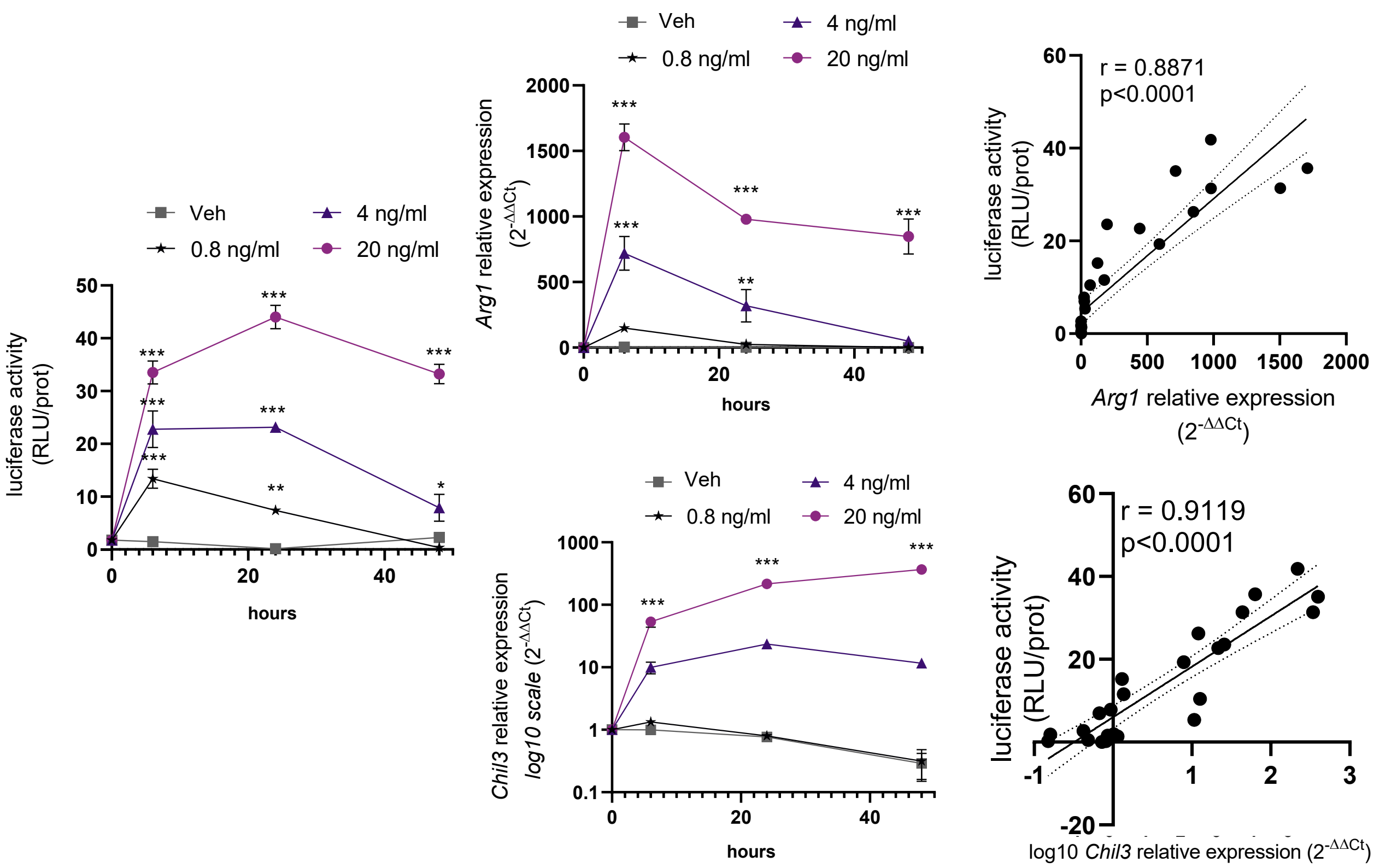

Figure S2. IL-4–induced responses in bone marrow–derived macrophages. Bone marrow–derived macrophages were treated with IL-4 at the indicated doses (0, 0.8, 4, 20 ng/mL) for 0, 6, 24, or 48 h. Luciferase activity in protein extracts is shown as RLU per µg protein (mean ± SEM). Relative expression of *Arg1* and *Ym1* (*Chil3*) is shown (*Arg1*, upper panels; *Ym1*, lower panels; mean ± SEM). Statistical analysis was performed using two-way ANOVA with Dunnett's test versus vehicle (n = 2). Correlation between luciferase activity and gene expression is shown (right); Pearson's r and p-values are reported.

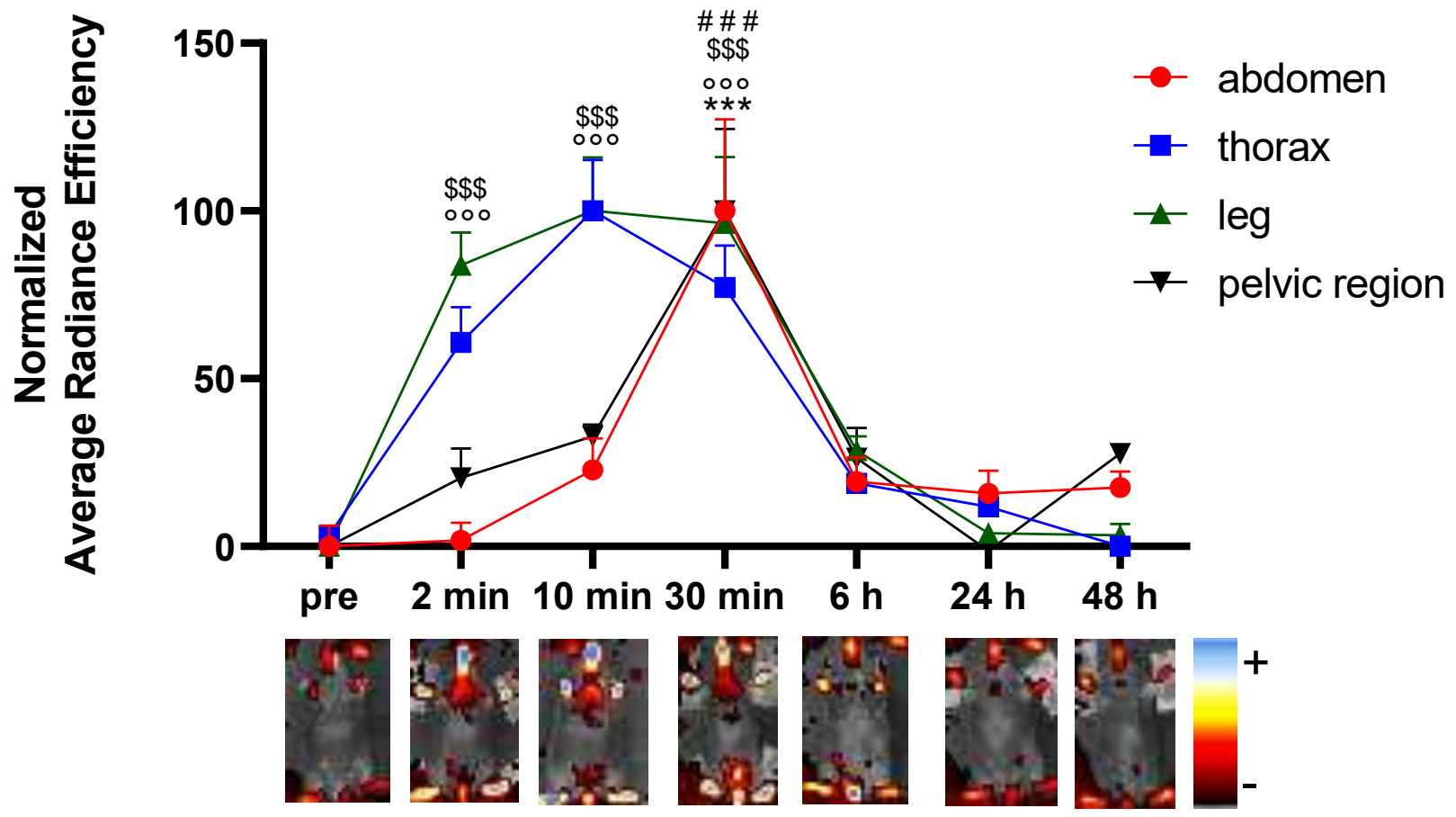

Figure S3. **Kinetic profile of fluorescently labelled mRNA-LNPs *in vivo* in different body districts**. C57BL/6 mice were intravenously injected with 10 µg of Cy5.5-labelled m¹Ψ-modified mRNA. Fluorescence was monitored at serial time points by *in vivo* imaging. Normalized fluorescence signals are presented as the mean ± SEM per mouse for each specified region of interest (ROI). For each body district, the highest detected signal has been placed at 100%. Representative *in vivo* images are shown. Statistical analysis was performed using two-way ANOVA followed by Dunnett's multiple comparisons test versus time zero (pre). $$$ $p < 0.001$ (leg); °°° $p < 0.001$ (thorax); *** $p < 0.001$ (abdomen); ### $p < 0.001$ (pelvic region).

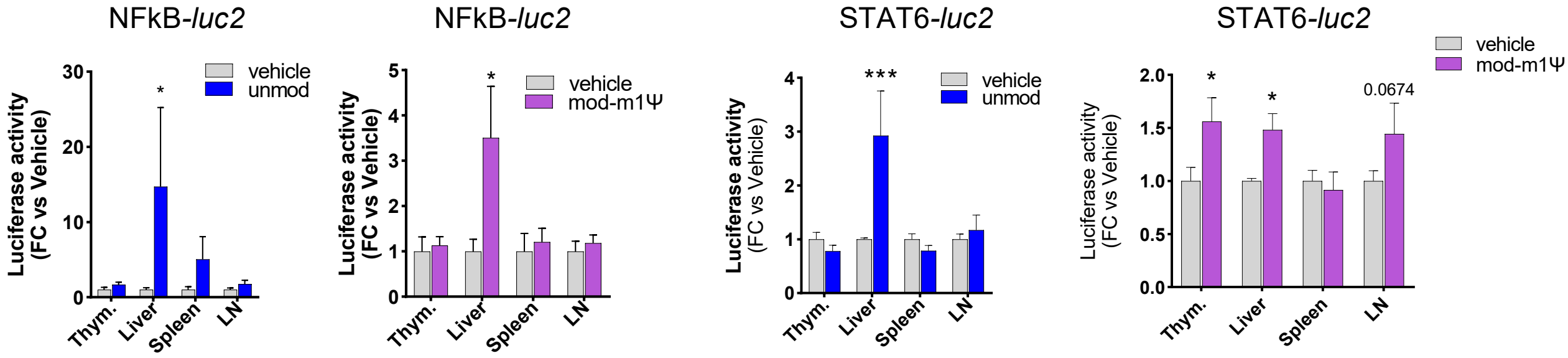

Figure S4. Bioluminescence in immune organs 5 hours after systemic vaccine administration. Bioluminescence emission from selected immune organs is shown 5 h after treatment. Quantification (derived from Figure 5A) is presented as mean photon emission (photons/s/cm²/sr) normalized to vehicle ± SEM. Statistical analysis was performed using two-way ANOVA with Dunnett's test versus vehicle (*p < 0.05, ***p < 0.001). Thym, thymus; LN, inguinal lymph nodes (right and left).

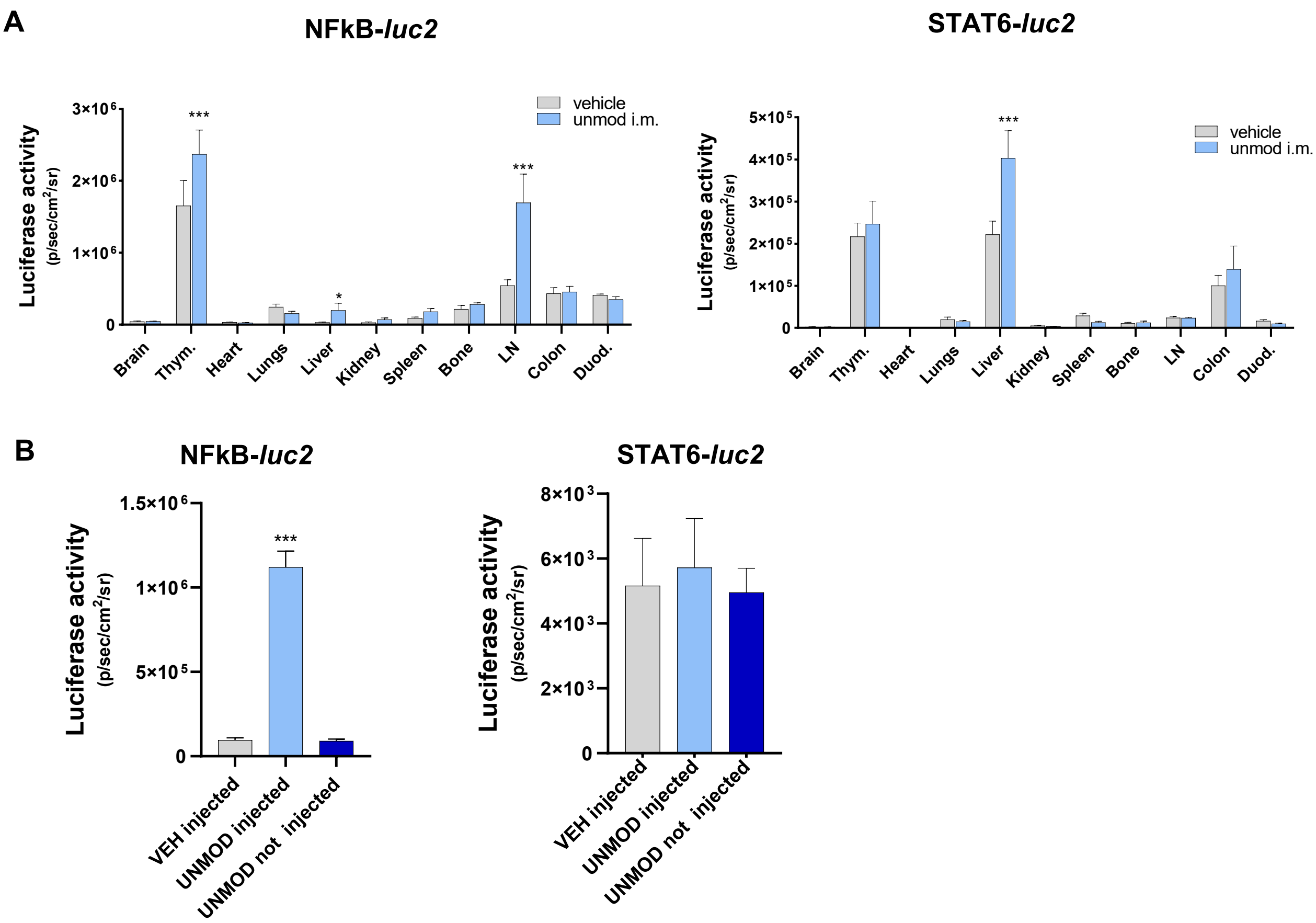

Figure S5. Ex vivo bioluminescence 5 hours after intramuscular administration of mRNA-LNP vaccine. Quantification of bioluminescence in explanted organs from NFκB-*luc2* and STAT6-*luc2* reporter mice 5 h after treatment with unmodified vaccine (10 µg) or vehicle (glucose solution). **(A)** Data are presented as mean photon emission (photons/s/cm²/sr) ± SEM (n = 6). Statistical analysis was performed using two-way ANOVA with Dunnett's test versus vehicle (*p < 0.05, **p < 0.01, ***p < 0.001). Thym, thymus; LN, inguinal lymph nodes (right and left); Duod., duodenum. **(B)** Bioluminescence signal in injected and contralateral (non-injected) muscle. Data are presented as mean photon emission (photons/s/cm²/sr) ± SEM (n = 3). Statistical analysis was performed using one-way ANOVA with Dunnett's test versus vehicle (***p < 0.001).

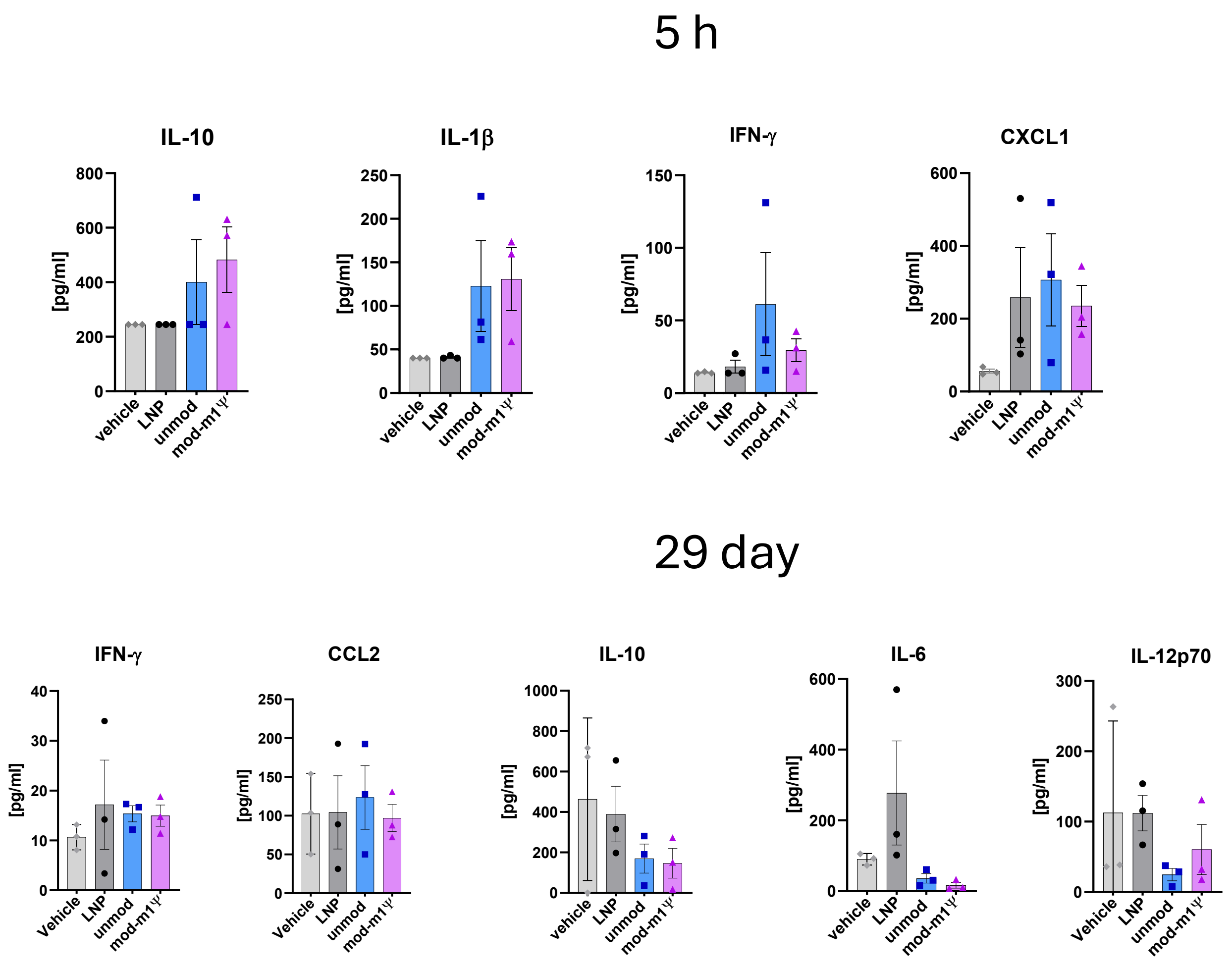

Figure S6. Plasma cytokine levels. Plasma cytokine levels at 5 hours and 29 days post-administration are shown. Data are presented as mean ± SEM (n = 3). Statistical analysis was performed using one-way ANOVA with Dunnett's test versus vehicle; no significant differences were detected. LNP: empty lipid nanoparticles; unmod: unmodified vaccine; mod-m1Ψ: N1-methylpseudouridine-modified vaccine.

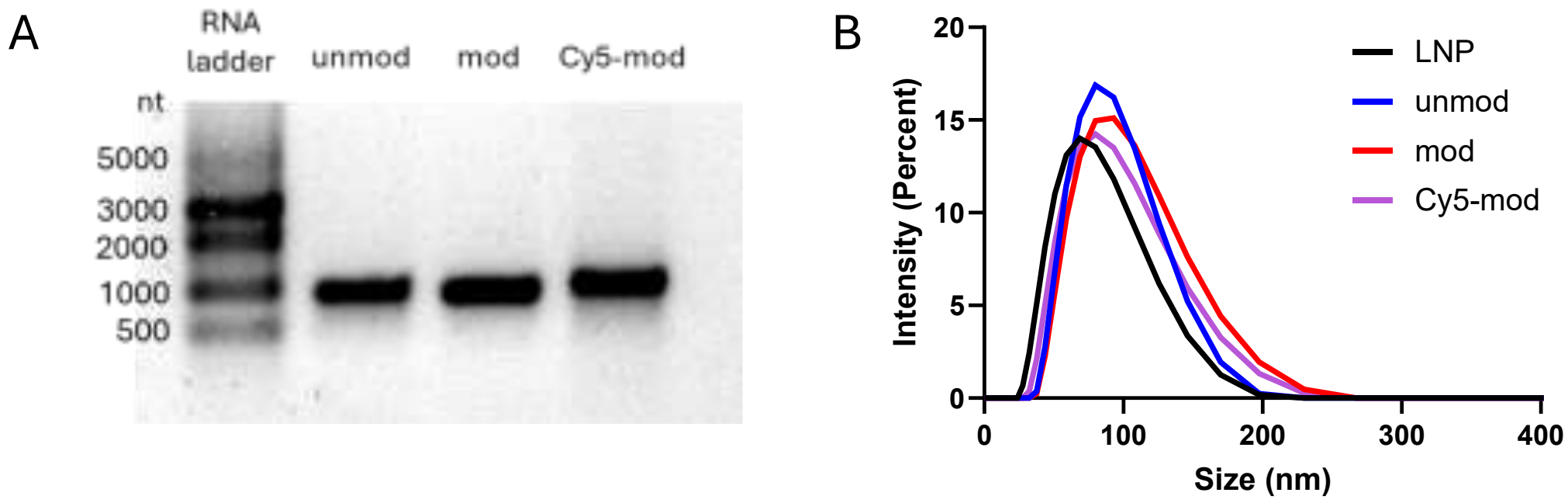

C

Formulation	Z-average (nm)	PDI	Zeta potential (mV)	EE(%)
**LNP**	70.22	0.2132	-2.525	N/A
**unmod**	82.96	0.1773	-6.255	93.2
**mod**	85.13	0.1111	-7.425	92.6
**Cy5-mod**	75.64	0.1459	-7.18	91.8

Figure S7. Physicochemical characterization of RNA-loaded lipid nanoparticles. (A) Agarose gel electrophoresis of synthesized mRNAs. An RNA ladder (nt) is shown alongside unmodified mRNA (unmod), N1-methylpseudouridine-modified mRNA (mod), and Cy5-labelled modified mRNA (Cy5-mod). **(B)** Size distribution profiles measured by dynamic light scattering (DLS) for LNPs encapsulating different RNA constructs. Curves correspond to empty LNPs (LNP, black), unmodified mRNA (unmod, blue), modified mRNA (mod, red), and Cy5-modified mRNA (Cy5-mod, purple). **(C)** Summary of physicochemical properties of LNPs formulations, including Z-average diameter, polydispersity index (PDI), zeta potential, and encapsulation efficiency (EE%).
